# Lipid nanoparticle mRNA delivery preserves CAR T cell cytotoxicity and limits exhaustion compared to electroporation

**DOI:** 10.1016/j.omtn.2026.102929

**Published:** 2026-04-28

**Authors:** Samira Picht, Martí Farrera-Sal, Anna L. Hiller, Sophia Brumhard, Antonia M. Klaas, Sarah Schulenberg, Rebecca Friedrich, Cedric Scholz, Lisa Hemmerling, David N. Simon, Gerhard Krönke, Chantal Pichon, Leif E. Sander, Hans-Dieter Volk, Manfred Gossen, Michael Schmueck-Henneresse, Norman M. Drzeniek

**Affiliations:** 1 Berlin Institute of Health (BIH) at Charité – Universitätsmedizin Berlin, BIH Center for Regenerative Therapies (BCRT), Experimental Immunotherapy, Augustenburger Platz 1, 13353 Berlin, Germany; 2 Charité - Universitätsmedizin Berlin, Corporate Member of Freie Universität Berlin and Humboldt-Universität zu Berlin, Department of Infectious Diseases, Respiratory and Critical Care Medicine, Charitéplatz 1, 10117 Berlin, Germany; 3 Berlin Institute of Health (BIH) at Charité, Charité - Universitätsmedizin Berlin, Berlin, Germany; 4 Department of Rheumatology and Clinical Immunology at Charité - Universitätsmedizin Berlin, Charitéplatz 1, 10117 Berlin, Germany; 5 Deutsches Rheuma-Forschungszentrum Berlin, Charitéplatz 1, 10117 Berlin, Germany; 6 Department of Internal Medicine 3, University of Erlangen-Nuremberg and Universitätsklinikum Erlangen, Ulmenweg 18, 91054 Erlangen, Germany; 7 Deutsches Zentrum für Immuntherapie (DZI), University of Erlangen-Nuremberg and Universitätsklinikum Erlangen, Ulmenweg 18, 91054 Erlangen, Germany; 8 ART ARNm US 55 Inserm, LI2RSO University of Orléans, 45100 Orléans, France; 9 Institut Universitaire de France, 1 rue Descartes, 75035 Paris, France; 10 Charité - Universitätsmedizin Berlin, Corporate Member of Freie Universität Berlin and Humboldt-Universität zu Berlin, Institute of Medical Immunology, Augustenburger Platz 1, 13353 Berlin, Germany; 11 Institute of Active Polymers, Helmholtz-Zentrum Hereon, Kantstraße 55, 14513 Teltow, Germany

**Keywords:** MT: delivery strategies, CAR t cells, mRNA electroporation, lipid nanoparticles, autoimmune disease, transient gene expression, chemotaxis, exhaustion markers, multiplexed engineering

## Abstract

Chimeric antigen receptor (CAR) T cells offer a promising strategy for the treatment of autoimmune diseases. However, clinical translation is limited by the high cost, complexity, and poor scalability of current manufacturing, restricting broad patient access and persisting safety concerns including insertional mutagenesis risk from integrating vectors and uncontrolled long-term CAR T cell persistence. *In-vitro*-transcribed (IVT) mRNA enables transient, non-integrating CAR expression with improved safety and scalability, making it particularly suited for non-malignant indications where prolonged persistence may not be required. Here, we systematically compare two IVT mRNA delivery platforms, electroporation and lipid nanoparticles (LNPs), for transient CAR T cell engineering in primary human T cells using single-cell transcriptomics and functional cell assays. We show that electroporation yields higher transfection efficiency and more sustained CAR surface expression, whereas LNP delivery reduces stress- and senescence-related transcriptional signatures as well as exhaustion marker expression, while enhancing antigen-driven activation, chemotactic responses, and cytotoxic function. Our comparative analysis highlights that the mode of mRNA delivery is associated with distinct transcriptional signatures and functional properties of CAR T cells, providing a framework to guide future development of mRNA-based approaches. These insights support LNP-mediated delivery as a functionally favorable strategy for transient CAR T cell engineering in autoimmune disease and beyond.

## Graphical abstract

**Figure:**
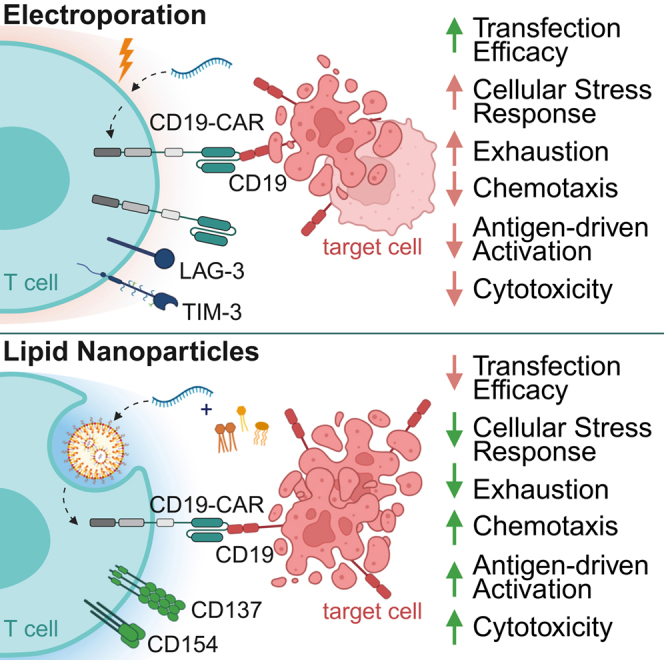

## Teaser

A direct comparison of electroporation and lipid nanoparticle (LNP) delivery for IVT mRNA CAR T cell engineering reveals distinct transcriptional and functional programs. Electroporation induces stress-associated signatures, whereas LNP delivery preserves T cell fitness, limits exhaustion, and maintains cytotoxicity and migration, informing development of transient CAR T cell strategies for autoimmune disease.

## Introduction

Chimeric antigen receptor (CAR) T cells have shown transformative efficacy in both B cell malignancies^1^^,^^2^ and, more recently, autoimmune diseases,^3^ such as systemic lupus erythematosus,^4^^,^^5^ vasculitis,^6^^,^^7^ and rheumatoid arthritis,^8^^,^^9^ enabling immune reset and durable drug-free remission, representing a potentially game-changing curative approach.^10^ However, the broader application of CAR T cell therapy in autoimmunity faces distinct translational barriers,^11^^,^^12^^,^^13^^,^^14^ including limited controllability of CAR T cell persistence necessitating inpatient monitoring,^15^^,^^16^^,^^17^ absence of readily available on-site products,^10^ potential of insertional mutagenesis from integrating vectors,^18^ and poor scalability of autologous manufacturing.^19^^,^^20^ Although cancer CAR T cell therapies require sustained persistence for efficacy,^21^^,^^22^ autoimmune indications may not require long-term CAR T cell persistence.^23^ Clinical data from us and others demonstrate that CAR T cells can induce durable immune reset despite transient CAR T cell persistence,^4^^,^^5^^,^^7^^,^^9^^,^^24^^,^^25^ highlighting the opportunity for safe, reversible engineering platforms, tailored to autoimmunity. *In-vitro*-transcribed (IVT) mRNA offers a promising platform, combining transient, non-integrating mode of action with rapid protein expression and minimal cellular footprint.^26^^,^^27^^,^^28^^,^^29^^,^^30^^,^^31^^,^^32^ Moreover, mRNA’s broad non-toxic dose range in T cells,^27^^,^^33^ enables co-expression of multiple functional proteins,^32^ allowing to leverage additional T cell functions beyond antigen recognition, such as migration and infiltration of tissues. CAR T cells effectively traffic to lymphoid and inflamed tissues, enabling deeper B cell depletion than B cell targeting biologics in autoimmune disease.^7^^,^^24^^,^^34^ Exploiting chemokine ligand-receptor systems^35^ to actively modulate T cell migration may further enhance potency, precision, reduce systemic toxicity, and lower the required cell dose. As an illustrative model, we target CAR T cell homing to secondary lymphoid organs through co-expression of CCR7 and the CAR. CCR7 overexpression may additionally promote central memory-like T cell phenotypes, associated with improved persistence and functionality,^36^^,^^37^ particularly relevant given reduced abundance in elderly patients, limiting product quality in autologous settings.^38^ These considerations underscore the potential of mRNA-based CAR T cell engineering to fine-tune T cell function, trafficking, and phenotype, and the need to elucidate the comparative effects of mRNA delivery strategies on T cell functionality.

Electroporation remains the gold standard for IVT mRNA delivery into primary human T cells for *in vitro* and *ex vivo* applications.^39^^,^^40^^,^^41^^,^^42^ However, electroporation requires transient membrane permeabilization to introduce naked mRNA, causing viability loss, physical stress, and potential reduction in subsequent T cell fitness.^43^ Alternatively, lipid nanoparticles (LNPs) have emerged as an *ex vivo* mRNA delivery platform.^31^^,^^44^^,^^45^ By encapsulating mRNA and relying on endocytic uptake, LNPs avoid direct membrane disruption and may reduce cellular stress.^28^^,^^44^^,^^46^^,^^47^ Although LNPs are postulated to better preserve T cell function, mechanistic understanding and actual functional benefits for CAR T cell potency and T cell function in general remain poorly understood.^45^ Comprehensive systematic comparisons of electroporation and LNP-mediated delivery in primary human T cells remain limited. Previous reports have suggested differences in functional potency and expression kinetics with nanocarrier-based delivery,^45^ but these parameters have not been evaluated side by side with electroporation under controlled conditions. Here, we directly compare both approaches, assessing transfection efficiency, expression kinetics, functional activity, exhaustion, and transcriptomic profiles to provide mechanistic and translational insights. To evaluate multiplexing capacity and trafficking-related phenotypes, we co-delivered CCR7 and CXCR3 IVT mRNAs as model targets. Together, these investigations provide foundational insight into phenotypic and functional consequences of distinct IVT mRNA delivery strategies, informing *ex vivo* engineering and future *in vivo* applications of CAR T cell therapy in autoimmunity.

## Results

### IVT mRNA multiplexing enables efficient multi-protein expression in CAR T cells

**Figure 1:**
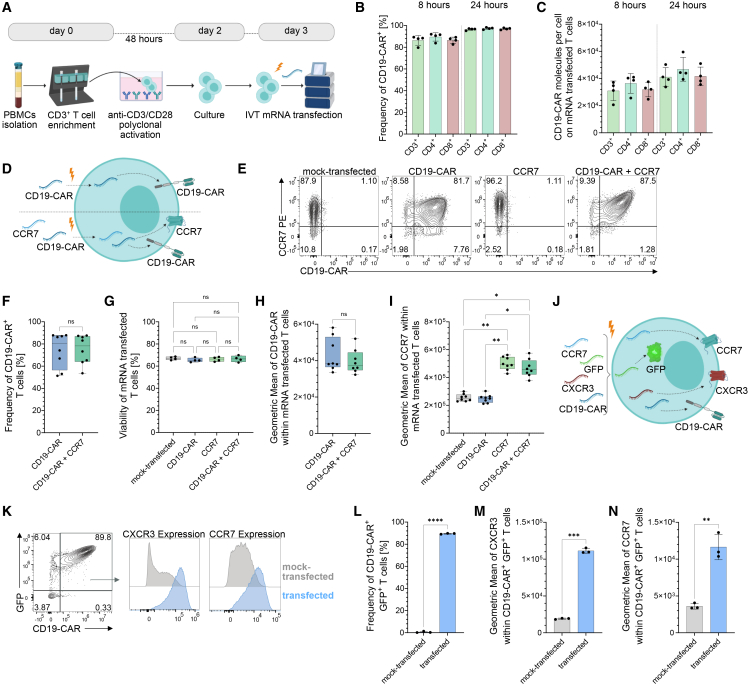
Transfection efficacy of CD19-CAR mRNA and co-expression of multiple IVT mRNAs in polyclonally activated T cells (A) Experimental workflow: PBMCs from healthy donors were isolated, CD3^+^ T cells enriched by MACS, polyclonally activated with plate-bound anti-CD3/CD28 antibodies for 48 h, and electroporated with IVT mRNA on day 3. Created with BioRender.com. (B and C) Flow cytometric analysis of CD19-CAR^+^ frequency (B) and CD19-CAR molecules per cell (C) in CD3^+^, CD4^+^, and CD8^+^ T cell subsets at 8 h and 24 h post-electroporation. (D) Schematic of single and co-transfection approach using CD19-CAR ± CCR7 mRNA. Created with BioRender.com. (E) Representative plots of mock-transfected control, CD19-CAR-only, CCR7-only, and CD19-CAR + CCR7 co-transfected T cells, showing CD19-CAR and CCR7 expression 8 h post-transfection. (F) Viability of T cells following single and dual mRNA transfection, assessed by live/dead staining 8 h post-transfection. (G–I) Quantification of CD19-CAR-only versus CD19-CAR + CCR7 co-expression: CD19-CAR^+^ frequency (G), CD19-CAR geometric mean fluorescence intensity (H), and CCR7 geometric mean fluorescence intensity (I) in CD3^+^ T cells, 8 h after mRNA transfection (*n* = 8 donors). (J) Schematic of multiplexing approach using four IVT mRNAs (CD19-CAR, CCR7, CXCR3, and GFP). Created with BioRender.com. (K–N) Flow cytometric analysis of four-mRNA multiplexing showing frequency of CD19-CAR^+^ GFP^+^ double-positive cells (K and L) and geometric mean fluorescence intensity of CXCR3 (M) and CCR7 (N), 8 h post-transfection (*n* = 3 donors). Statistical analysis between subpopulations was performed by one-way ANOVA with Tukey’s post hoc test or paired *t* test. ns, not significant; ∗*p* < 0.05, ∗∗*p* < 0.01, ∗∗∗*p* < 0.001, ∗∗∗∗*p* < 0.0001. Data represent mean ± SD from the indicated number of independent donors.

We established an effective IVT mRNA-based CAR T cell engineering platform using electroporation in polyclonally activated human T cells (Figure 1A). Following peripheral blood mononuclear cell (PBMC) isolation and CD3^+^ enrichment via magnetic-activated cell sorting (MACS), T cells were stimulated for 48 h via plate-bound anti-CD3/CD28 antibodies. On day 3, IVT mRNA was electroporated into the activated T cells using the Lonza 4D-Nucleofector system. Program EO115 was selected after systematic evaluation of six electroporation programs based on optimal T cell viability and CD19-CAR^+^ T cell transfection efficacy (Figures S1A and S1B). All programs tested resulted in comparable memory phenotype distribution (naive like [T_naive like_]: CD45RA^+^CCR7^+^, central memory [T_CM_]: CD45RA^–^CCR7^+^, effector memory [T_EM_]: CD45RA^–^CCR7^–^, and terminal effector memory [T_EMRA_]: CD45RA^+^CCR7^–^), with CD19-CAR T cell consisting predominantly of naive like and T_CM_s (Figure S1C). Flow cytometric analysis confirmed consistently high transfection efficiencies (>85% CD19-CAR^+^ T cells at 8 h, >95% at 24 h post-transfection) across tested mRNA concentrations (1–3 μg), indicating robust delivery with a modest dose-response effect 24 h post-transfection (Figure S1D). CAR expression was uniformly achieved in CD3^+^, CD4^+^, and CD8^+^ T cell subsets (>80% at 8 h; >95% at 24 h; Figure 1B), with protein expression reaching up to approximately 5 × 10^4^ CD19-CAR molecules per cell at 24 h (Figure 1C). These results demonstrate high efficiency and reproducibility of CAR expression across major T cell subsets. Limitations of stable genomic integration can be circumvented via IVT mRNA multiplexing enabling transient, combinatorial expression of CARs and accessory proteins to fine-tune T cell phenotype and function, improving quantitative control by avoiding genomic integration.^48^ To evaluate the potential of this strategy for complex CAR T cell programming, we initially co-transfected CD19-CAR and CCR7 IVT mRNAs into activated human T cells (Figure 1D). Both transcripts were efficiently expressed with preserved transfection efficiency (Figures 1E and 1F) and viability (∼70% viability at 24 h; Figure 1G). Co-transfection yielded a median expression of 78.20% CD19-CAR^+^ T cells (range 53.60%–87.60%; Figure 1F), comparable to single-transfected controls, with similar CAR surface expression intensity (Figure 1H). Surface expression of CCR7 was significantly increased in CCR7-transfected cells (Figure 1I), with a peak observed at 8 h post-transfection (Figure S1E). To demonstrate multiplexing scalability, we next co-delivered 4 distinct IVT mRNAs encoding CD19-CAR, CCR7, CXCR3, and GFP (Figure 1J). No evidence of translational competition was observed (Figure 1K). At 8 h post-transfection, approximately 90% of T cells co-expressed CD19-CAR and GFP (Figures 1K and 1L), while CCR7 and CXCR3 were also robustly upregulated (Figures 1M and 1N), confirming co-delivery of multiple transcripts and functional co-expression. Concurrently, co-transfection with CXCR3 and CCR7 mRNAs significantly upregulated the respective homing markers (Figures 1M and 1N), confirming robust multi-protein expression relevant to functional trafficking modulation. Expression kinetics varied by protein, with GFP persisting for ≥4.5 days, CD19-CAR for ∼3.5 days, and chemokine receptors CCR7 and CXCR3 detectable for ∼24 h (Figures S1F–S1H). Together, these findings confirm that IVT mRNA multiplexing via electroporation enables transient, high-efficiency, multi-protein expression in primary human T cells, supporting the generation of customizable CAR T cell products without permanent genomic modification.

### CCR7 IVT mRNA co-delivery improves lymphatic tissue-associated chemotaxis and functionality of mRNA-engineered CD19-CAR T cells

**Figure 2:**
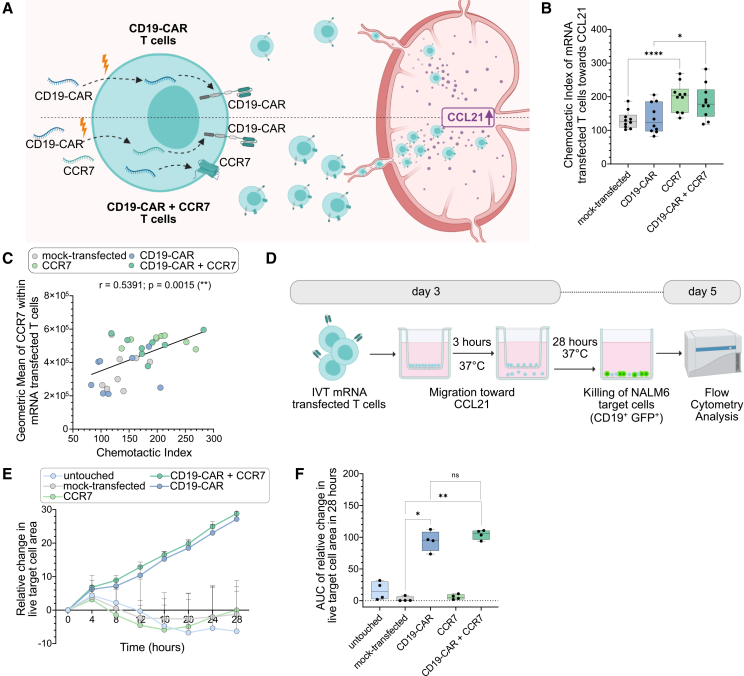
Co-transfection of CCR7 with CD19-CAR T cells enhances migration toward a lymph node-associated chemokine (A) Schematic showing the concept of improving CD19-CAR T cell migration toward CCL21 by co-transfecting CCR7 mRNA alongside CD19-CAR mRNA (lower) compared to conventional CD19-CAR T cells (upper). Created with BioRender.com. (B) Chemotactic index toward CCL21 for mock-transfected, CCR7-only, CD19-CAR-only, and CD19-CAR + CCR7 co-transfected T cells. The chemotactic index was calculated as the ratio of migrated cells in the presence versus absence of CCL21, quantified by flow cytometry. (C) Correlation between CCR7 expression level and migration. Scatterplot showing the relationship between CCR7 geometric mean fluorescence intensity and chemotactic index in CD19-CAR T cells. Pearson’s r and *p* values are indicated. (D) Experimental workflow: single- or co-transfected T cells were assessed in an *in vitro* transwell assay, first migrating toward CCL21 for 3 h, followed by co-culture with NALM6 target cells (CD19^+^ and GFP^+^). Killing efficiency was monitored over 28 h with 4-h intervals using live-cell imaging. Created with BioRender.com. (E) Killing kinetics after CCL21-mediated migration. Time-course analysis of cytotoxicity against NALM6 target cells by untouched, mock-transfected, CCR7-only, CD19-CAR-only, and CD19-CAR + CCR7 co-transfected T cells over 28 h. Data represent mean ± SD from *n* = 4 independent donors. (F) Area under the curve (AUC) analysis of total killing capacity over the 28 h observation period for the same groups. Data represent mean ± SD from *n* = 4 independent donors. Statistical analysis was performed using one-way ANOVA with Tukey’s post hoc test (B) or Friedman test followed by Dunn’s multiple comparison test (F). ns, not significant; ∗*p* < 0.05, ∗∗*p* < 0.01, ∗∗∗*p* < 0.001, ∗∗∗∗*p* < 0.0001. Data represent mean ± SD from *n* = 8 independent donors, unless stated otherwise.

To demonstrate the functional relevance of IVT mRNA-based multiplexing in tailoring CAR T cell migration for autoimmune indications, we evaluated whether co-expression of the lymph node-homing receptor CCR7 enhances the ability of CD19-CAR T cells to migrate toward CCL21, a chemokine that mediates lymphoid tissue localization^49^^,^^50^ (Figure 2A). This is particularly relevant in autoimmune disease, where autoreactive B cells primarily reside in secondary lymphoid organs. To mimic tissue-directed trafficking and evaluate potential functional gains, we assessed CCR7-mediated migration using two complementary approaches. First, we performed a 3-h transwell migration assay with CD19-CAR T cells (±CCR7 mRNA) (Figures 2B and 2C). Second, we designed a two-step assay combining *in vitro* migration and cytotoxicity: CD19-CAR T cells (±CCR7 mRNA) were placed in a transwell system to assess migration toward CCL21 over 3 h, followed by live-cell imaging of cytotoxicity of migrated CAR T cells against NALM6 target T cells (CD19^+^ GFP^+^) over 28 h in the lower chamber (Figure 2D). Quantitative analysis revealed that CCR7-only-transfected T cells and CD19-CAR + CCR7 co-transfected T cells both showed significantly enhanced migration toward CCL21 compared to unmodified controls (Figure 2B). Co-transfected T cells maintained CCR7-mediated chemotaxis, confirming that dual mRNA delivery does not impair individual protein function. Moreover, CCR7 surface expression correlated positively with the chemotactic index toward CCL21, indicating a dose-dependent relationship between receptor density and migratory capacity (Figure 2C). During live-cell cytotoxicity assays, co-transfected T cells retained their enhanced migratory advantage and showed a trend toward improved killing of NALM6 target cells in the presence of CCL21 (Figures 2E and 2F), though with inter-donor variability in the magnitude of this effect (Figure S2A). In the absence of CCL21, both migration and subsequent target-cell killing were minimal (Figure S2B), confirming the functional relevance of the CCR7-CCL21 axis in this model. To further highlight the therapeutic potential of CCR7 co-transfection, particularly to compensate for reduced central memory T cell abundance in elderly patients,^38^ we examined CCR7 mRNA transfection in T cells with different baseline CCR7 expression. IVT mRNA encoding CCR7 and CD19-CAR was successfully co-transfected in both endogenous CCR7-expressing T cells (>65% at 4 h) and CCR7 knock-out T cells (>42% at 4 h; Figure S2C). Functional comparison of both sample groups revealed distinct patterns in their cytotoxic capacity against NALM6 target cells (CD19^+^ GFP^+^) over 39 h, following a CCL21-mediated CD19-CAR T cell migration (Figures S2D and S2E). CD19-CAR T cells in which endogenous CCR7 had been knocked out showed the lowest killing capacity, which could be effectively rescued by introducing CCR7 IVT mRNA, restoring killing capacity to levels comparable with endogenously CCR7-expressing T cells (Figures S2D and S2E). These data demonstrate that mRNA-based CCR7 co-expression effectively enhances functional migration of CAR T cells toward lymphoid chemokine gradients, without impairing cytotoxic capacity and can restore migratory and cytotoxic function even in T cells lacking CCR7. Transient multiplexed mRNA delivery thus allows combinatorial control of both killing specificity and tissue tropism, offering a modular strategy for improving CAR T cell functionality.

### Comparison of electroporation and LNP-mediated IVT mRNA delivery reveals distinct CAR expression profiles

**Figure 3:**
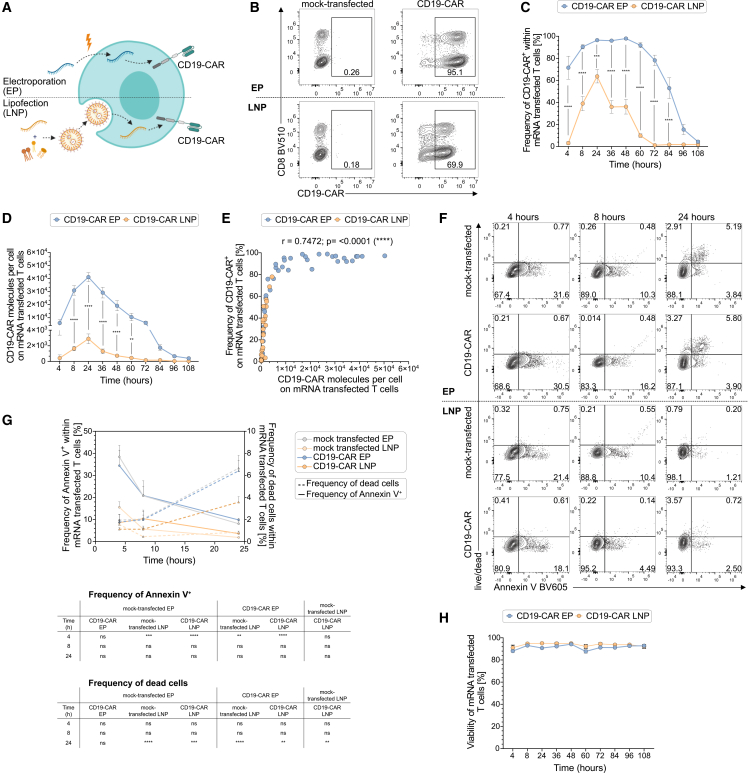
Comparison of CD19-CAR expression kinetics using electroporation versus lipid nanoparticle delivery (A) Schematic illustration of IVT mRNA delivery mechanisms: electroporation (EP)-mediated delivery of naked IVT mRNA through transient membrane permeabilization versus lipid nanoparticle (LNP)-encapsulated IVT mRNA uptake via endocytosis, both leading to CD19-CAR protein translation. (B) Representative flow cytometry plots showing CD19-CAR expression in mock-transfected and mRNA-transfected T cells 24 h post-transfection, for electroporation (upper) and LNP delivery (lower). (C and D) Flow cytometric analysis of CD19-CAR^+^ frequency (C) and CD19-CAR molecules per cell (D) over 96 h after mRNA delivery by electroporation or LNPs. (E) Correlation between CD19-CAR^+^ frequency and CD19-CAR molecules per cell. (F) Representative flow cytometry plots showing annexin V and live/dead staining in mock-transfected and CD19-CAR mRNA-transfected T cells 4, 8 and 24 h post-transfection, for electroporation (upper) and LNP delivery (lower). (G) Flow cytometric analysis of annexin V^+^ and dead cell frequency in mock-transfected and mRNA-transfected T cells (EP or LNP) over 24 h comparing electroporation and LNP-transfection as IVT mRNA delivery methods. (H) Flow cytometric analysis of T cell viability, assessed by live/dead staining over 96 h post-transfection. Statistical analysis for differences between groups was performed by two-way repeated-measures ANOVA with Šidák’s multiple-comparison test. ns, not significant; ∗*p* < 0.05, ∗∗*p* < 0.01, ∗∗∗*p* < 0.001, ∗∗∗∗*p* < 0.0001. Data represent mean ± SEM from *n* = 4 independent donors.

The choice of IVT mRNA delivery method critically influences CAR expression kinetics and therapeutic potential.^45^ To optimize IVT mRNA-based CD19-CAR delivery into primary human T cells, we performed a direct comparison of electroporation and LNP-mediated transfection (Figure 3A). Electroporation, the current standard for *ex vivo* T cell modification,^39^^,^^40^ enables direct cytoplasmic delivery and translation of naked mRNA via transient membrane permeabilization.^43^ In contrast, LNP’s mRNA payload is delivered to and translated in the cytoplasm only upon endosomal uptake and release, as well as LNP disintegration.^34^^,^^51^ They are associated with lower cytotoxicity and offer potential for *in vivo* use.^31^^,^^44^^,^^51^ We compared these two approaches head-to-head by evaluating transfection efficiency and CAR expression profiles following IVT mRNA delivery by electroporation versus LNPs. Electroporation achieved more than 95% CD19-CAR^+^ T cells at 24 h post-transfection, whereas LNP-mediated delivery reached approximately 65% (Figures 3B, 3C, and S3A). In addition to higher transfection frequency, electroporated T cells expressed substantially more CD19-CAR molecules per cell (∼4 × 10^4^ vs. ∼3 × 10^3^), as quantified by flow cytometry (Figure 3D). Both methods exhibited peak expression at 24 h, with electroporation sustaining detectable CAR levels for 4 days, compared to 3 days following LNP delivery (Figures 3C and 3D). Notably, in our experimental conditions, electroporation-mediated expression after 3 days exceeded the peak expression levels achieved by LNP transfection at 24 h post-transfection. Correlation analysis revealed a positive correlation between the frequency of CD19-CAR^+^ cells and the number of CD19-CAR molecules per cell. While high CD19-CAR^+^ frequencies (>90%) were rapidly reached following electroporation, CD19-CAR molecules per cell continued to vary even at this apparent saturation point. This indicates that CAR surface density represents an independent parameter beyond CD19-CAR^+^ frequency that may contribute to functional heterogeneity in CAR T cell products (Figure 3E). Electroporated T cells showed transiently elevated phosphatidylserine exposure on the cell surface compared to LNP-transfected T cells (Figures 3F, 3G, and S3B). This was not accompanied by cell death, as assessed by staining for intactness of the cell membrane (Figure 3H) and occurred independently of the presence of IVT mRNA, suggesting that the electroporation procedure itself (mock-transfected) induces transient cellular stress. These findings demonstrate that electroporation and LNP-mediated mRNA delivery exhibit distinct CD19-CAR expression profiles; electroporation achieved higher transfection efficiency and longer expression persistence than LNP-based approaches, but also induced transient stress.

### IVT mRNA delivery preserves early memory T cell phenotypes across subsets

**Figure 4:**
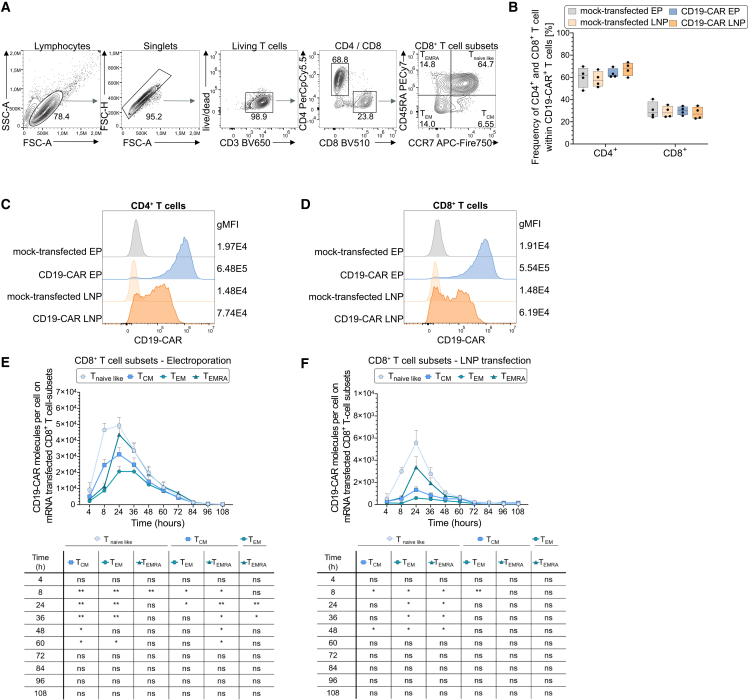
Memory phenotype characterization of IVT mRNA-transfected CD19-CAR T cells (A) Representative flow cytometry plots showing the gating strategy for CD8^+^ memory T cell subsets in primary human CD3^+^-enriched T cells. Subsets were defined by CD3^+^, CD4^–^, CD8^+^, and CCR7/CD45RA expression as follows: naive like (T_naive like_; CCR7^+^ CD45RA^+^), central memory (T_CM_; CCR7^+^ CD45RA^–^), effector memory (T_EM_; CCR7^–^ CD45RA^–^), and terminally differentiated effector (T_EMRA_; CCR7^–^ CD45RA^+^). (B) Frequencies of CD4^+^ and CD8^+^ T cells within CD19-CAR^+^ populations compared with mock-transfected controls after electroporation or LNP transfection. (C and D) Geometric mean fluorescence intensity (gMFI) of CD19-CAR expression in bulk CD4^+^ (C) and CD8^+^ (D) T cell populations following electroporation or LNP-mediated transfection; representative experiment. (E and F) CD19-CAR surface expression across memory T cell subsets within the CD8^+^ population following electroporation (E) or LNP-mediated (F) mRNA delivery over 108 h post-transfection. Statistical analysis was performed by one-way ANOVA with Tukey’s post hoc test (B) or by two-way repeated-measures ANOVA with Šidák’s multiple-comparison test (E and F). ns, not significant; ∗*p* < 0.05, ∗∗*p* < 0.01, ∗∗∗*p* < 0.001, ∗∗∗∗*p* < 0.0001. Data represent mean ± SD from *n* = 4 independent donors, unless stated otherwise.

To evaluate whether IVT mRNA delivery efficiency varies between T cell subsets and to assess differential susceptibility to transfection or mRNA translation capacity, we performed a detailed phenotypic characterization of CD19-CAR T cells following electroporation or LNP-mediated IVT mRNA delivery. Flow cytometry at 24 h post-transfection (Figure 4A) showed that CD4^+^ and CD8^+^ T cell frequencies among CD19-CAR^+^ cells were comparable to mock-transfected controls, with approximately 70% CD4^+^ and 30% CD8^+^ CAR T cells irrespective of the delivery method (Figure 4B). This indicates that neither electroporation nor LNPs introduce subset bias in transfection efficiency at the CD4/CD8 level. Quantitative analysis of CD19-CAR surface expression revealed substantially higher geometric mean fluorescence intensity in both CD4^+^ and CD8^+^ T cells transfected via electroporation compared to LNP delivery (Figures 4C and 4D). To further dissect subset-specific effects, T cells were classified based on CD45RA and CCR7 expression into T_naive like_ (CD45RA^+^CCR7^+^), T_CM_ (CD45RA^–^CCR7^+^), T_EM_ (CD45RA^–^CCR7^–^), and T_EMRA_ (CD45RA^+^CCR7^–^) subsets (Figure 4A). Across both CD4^+^ and CD8^+^ compartments, T_naive like_ and T_EMRA_ subsets exhibited the highest CD19-CAR molecule density per cell, independent of the delivery method, whereras T_EM_ cells consistently showed the lowest CAR expression (Figures 4E, 4F, S4A, and S4B). These results highlight distinct subset-specific transfection and expression efficiencies. To optimize LNP-based transfection, we compared T cells cultured in ImmunoCult versus CTL media. CTL medium improved LNP-mediated transfection efficiency, yielding 80%–90% CD19-CAR^+^ T cells compared to 60%–70% CD19-CAR^+^ T cells with ImmunoCult at 24 h post-transfection (Figure S4C). Immunophenotypic profiling revealed that both electroporated and LNP-transfected CD19-CAR T cells, irrespective of culture media, were enriched for early differentiated subsets, such as T_naive like_ and T_CM_ cells (Figure S4D), which are linked to long-term persistence and therapeutic efficacy.^36^^,^^37^^,^^52^^,^^53^ Despite lower CD19-CAR geometric mean fluorescence intensity in LNP-transfected cells, differentiation profiles were comparable between delivery methods. These findings support the notion that IVT mRNA-based delivery maintains early memory phenotypes relevant for therapeutic efficacy.

### Single-cell profiling reveals electroporation-induced stress and DNA damage signatures and LNP-associated effector programs

**Figure 5:**
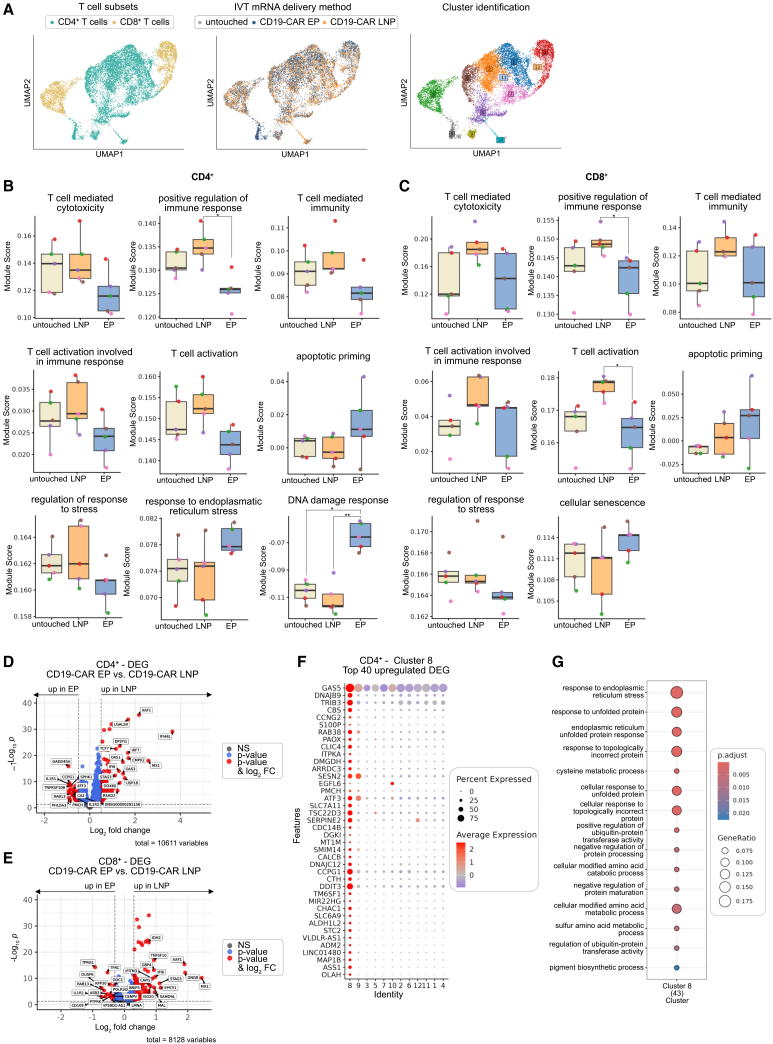
Single-cell transcriptional response of CD19-CAR T cells transfected with electroporation or LNP (A) UMAP embeddings of donor-integrated scRNA-seq data showing 10,883 high-quality transcriptomes annotated as CD4^+^ or CD8^+^ T cells, colored by T cell subtype, transfection method and leiden clustering. (B and C) Module scores of curated GO term gene sets in CD4^+^ (B) and CD8^+^ (C) T cell subsets. Median module scores per donor are plotted. (D and E) Volcano plot of differentially expressed genes (DEGs) between LNP and EP transfected CD4^+^ (D) and CD8^+^ (E) T cells. DE testing was performed using a paired pseudobulk approach using DESeq2. Datapoints for genes with baseMean >50 are plotted, log2FoldChange threshold set to 0.5 (CD4^+^) and 0.3 (CD8^+^), adjusted *p* value threshold set to 0.05. Top 15 DEG according to Log2FoldChange above both thresholds are labeled. (F) Dotplot of top 40 upregulated DEG of cluster 8 vs. all other cluster. DE testing was performed using a paired pseudobulk approach using DESeq2. Genes with baseMean >50 and adjusted *p* value <0.05 were ranked according to log2FoldChange. (G) GO enrichment analysis of the top upregulated DEG of cluster 8 vs. all other cluster. Genes with baseMean >50 and adjusted *p* value <0.05 were considered and top 50 according to log2FoldChange were used as input. (B and C) Boxplots showing median and lower and upper hinges correspond to the first and third quartiles. Upper and lower whisker extends from the hinge to the largest/smallest value no further than 1.5 ∗ IQR from the hinge. Data beyond the end of the whiskers are plotted individually. Statistical comparisons of median module scores across groups were performed using the Kruskal-Wallis test, followed by Dunn’s post hoc test with Holm adjustment for multiple testing. ns, not significant; ∗*p* < 0.05, ∗∗*p* < 0.01, ∗∗∗*p* < 0.001, ∗∗∗∗*p* < 0.0001. Data represents data from *n* = 5 independent donors.

To characterize transcriptional changes induced by the different IVT mRNA delivery methods, we performed targeted single-cell RNA sequencing (scRNA-seq) of untouched T cell and CD19-CAR T cells at 24 h post-transfection. To examine whether the two delivery methods generated distinct T cell populations, we applied uniform manifold approximation and projection (UMAP) analysis for dimensional reduction and cluster identification (Figure 5A). This analysis revealed an electroporation-specific cluster (cluster 8) within the CD4^+^ T cell compartment (Figure 5A). This cluster comprised 4.48%–16.2% of electroporated CD19-CAR T cells (Figure S5A). Pathway enrichment analysis across all clusters revealed distinct functional signatures between the two IVT mRNA delivery methods (Figures 5B and 5C): LNP-transfected CD19-CAR T cells exhibited transcriptome signatures associated with enhanced T cell-mediated cytotoxicity, increased T cell activation, and positive regulation of immune effector processes in both CD4^+^ and CD8^+^ T cell subsets (Figures 5B and 5C). In contrast, electroporation induced increased response to endoplasmic reticulum stress, enhanced apoptotic priming, altered regulation of stress response pathways and significantly increased DNA damage response-associated transcriptional signatures in CD4^+^ T cells (Figure 5B). CD8^+^ T cells showed analogous signatures, including reduced response to stress, increased apoptotic priming and signatures associated with cellular senescence (Figure 5C). Accordingly, electroporated CD19-CAR T cells displayed upregulation of cellular stress response genes, including *GADD45A*, *ATF3*, *CCPG1*, and *PHLDA3*^54^^,^^55^^,^^56^^,^^57^^,^^58^^,^^59^ compared to LNP-transfected CD19-CAR T cells and untouched T cells (Figures 5D, 5E, S5B, and S5C), while LNP-transfected CD19-CAR T cells primarily activated interferon-stimulated genes, including *IFI44L*, *MX1*, *IFI6*, *DDX60*, *RSAD2*, *XAF1*, *IRF7*, and *USP18*^60^^,^^61^ (Figures 5D, 5E, and S5C). To further characterize the electroporation-specific cluster 8, we analyzed the 40 most upregulated differentially expressed genes in cluster 8, which revealed enrichment of genes associated with stress response and DNA damage, including *TRIB3*, *DDIT3*, *CCPG1*, *SESN2*, *DNAJB9*, and *ATF3*^55^^,^^56^^,^^62^^,^^63^^,^^64^ (Figure 5F). Pathway enrichment analysis of cluster 8 demonstrated significant enrichment for response to endoplasmic reticulum stress, response to unfolded protein, endoplasmic reticulum unfolded protein response, and response to topologically incorrect protein and multiple metabolic stress pathways (Figure 5G). Additional enriched pathways included cellular response to decreased oxygen levels, positive regulation of ubiquitin-protein transferase activity, and regulation of ubiquitin-protein transferase activity, collectively indicating profound cellular stress and cellular imbalance. These findings indicate that electroporation is associated with cellular stress, DNA damage response-related and senescence-related transcriptional signatures, which are consistent with reduced functional fitness. In contrast, LNP transfection elicited a moderate but measurable innate immune response at transcript level, which was not associated with stress or apoptotic signatures, instead preserving and potentially enhancing effector T cell programs.

### Distinct activation signatures in CAR T cells following LNP versus electroporation-based IVT mRNA delivery

**Figure 6:**
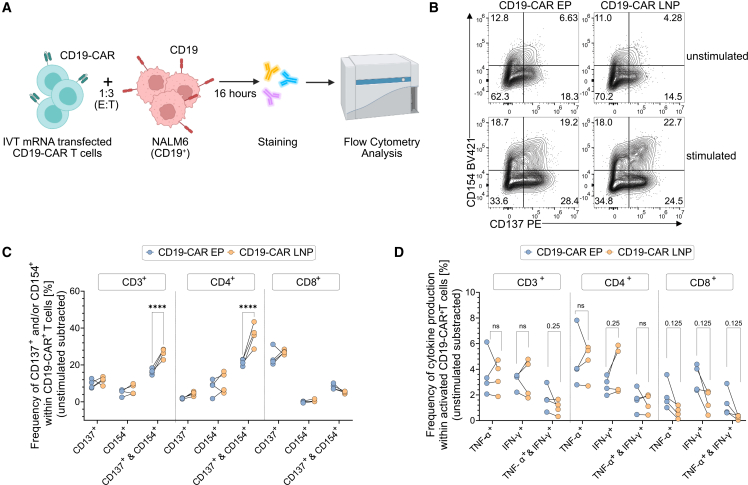
Functional characterization of CD19-CAR mRNA transfected T cells following antigen encounter (A) Experimental workflow of the overnight stimulation assay: mock-transfected and mRNA-transfected CD19-CAR T cells were stimulated with NALM6 (CD19^+^) cells for 16 h in the presence of brefeldin A to capture intracellular cytokine production by flow cytometry. Created with BioRender.com. (B) Representative flow cytometry plots showing activation marker expression (CD137 and CD154) in CD3^+^ T cells with (stimulated) and without (unstimulated) target cell stimulation. Comparisons are shown for electroporated (left) and LNP-transfected (right) CD19-CAR T cells. (C) Frequencies of activated (CD137^+^ and/or CD154^+^) CD3^+^, CD4^+^, and CD8^+^ T cell populations, comparing electroporation and LNP delivery methods. Data are background-subtracted. (D) Intracellular effector cytokine production (IFN-γ and TNF-α) within activated CD3^+^, CD4^+^, and CD8^+^ T cell populations. Data showing the relative increase in cytokine-producing T cells for LNP transfection normalized to electroporation. Statistical analysis was performed by paired *t* test. ns, not significant; ∗*p* < 0.05, ∗∗*p* < 0.01, ∗∗∗*p* < 0.001, ∗∗∗∗*p* < 0.0001. Data represent mean ± SD from *n* = 4 independent donors.

To determine how these transcriptional differences translate into functional consequences, we next examined CAR T cell function after antigen engagement. We analyzed activation markers and intracellular effector cytokine profiles of CD19-CAR T cells generated via electroporation or LNP transfection. CAR T cells were co-cultured with CD19-expressing NALM6 target cells for 16 h, followed by flow cytometric assessment of activation markers and intracellular cytokine production (Figure 6A). Electroporated CAR T cells showed elevated baseline expression of CD154 (CD40L) and CD137 (4-1BB), even in the absence of antigen (Figures 6B and S6A), indicating electroporation-induced non-specific activation, as demonstrated by elevated baseline activation in mock-transfected controls (Figure S6B). In contrast, LNP-transfected CAR T cells exhibited minimal activation under resting conditions but responded robustly upon antigen encounter, particularly within the CD4^+^ subset (Figures 6B, 6C, and S6C). Intracellular cytokine analysis revealed delivery-method-dependent functional signatures. CD4^+^ CAR T cells transfected via LNPs tend to produce more interferon-γ (IFN-γ) upon target cell stimulation (Figures 6D, S6D, and S6E), indicating strong antigen-specific activation. Electroporated CD8^+^ CAR T cells showed elevated tumor necrosis factor-α (TNF-α) production, a tendency toward increased IFN-γ production and higher frequencies of IFN-γ^+^/TNF-α^+^ double-positive cells, reflective of a potent pro-inflammatory cytokine response (Figures 6D and S6E). These findings demonstrate that while electroporation induces stronger baseline activation and effector cytokine production, LNP-mediated delivery promotes more specific, antigen-driven responses with lower background activation, features that may enhance safety and therapeutic control in clinical applications.

### LNP-transfected CAR T cells demonstrate superior functional fitness despite lower CAR expression

**Figure 7:**
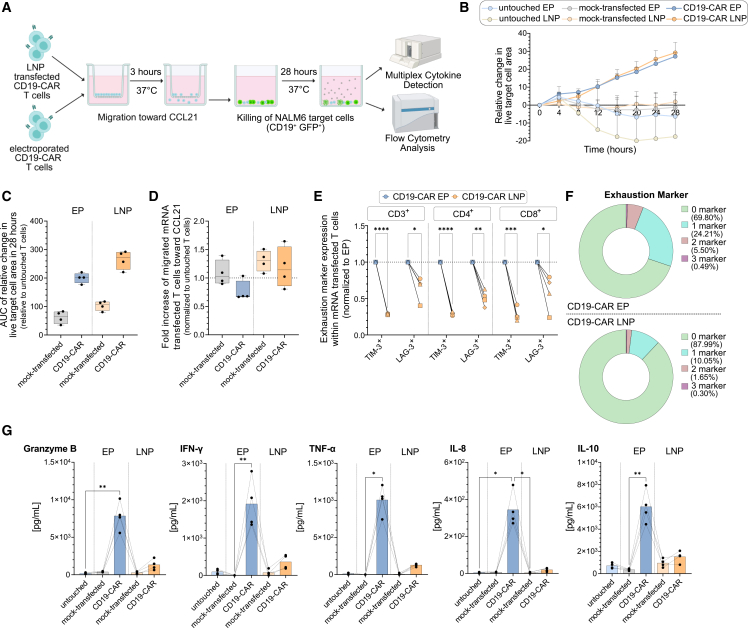
Functional characterization of CD19-CAR T cells produced by LNP-mediated versus electroporation-based mRNA delivery (A) Experimental workflow: electroporated and LNP-transfected T cells were assessed in an *in vitro* transwell assay, first migrating toward CCL21 for 3 h, followed by co-culture with NALM6 target cells (CD19^+^ GFP^+^). Killing efficiency was monitored over 28 h with 4-h intervals using live-cell imaging. Created with BioRender.com. (B) Killing efficacy of NALM6 (CD19^+^ GFP^+^) target cells using untouched, mock-transfected and CD19-CAR mRNA transfected T cells (EP and LNP) over 28 h assessed using live-cell imaging every 4 h. (C) Area under the curve (AUC) analysis of total killing capacity over the 28-h observation period for the same groups relative to untouched T cells. (D) Fold increase of migrated CD3^+^ T cells toward CCL21 chemokine normalized to untouched T cells comparing LNP and electroporation delivery methods for mock-transfected and CD19-CAR mRNA transfected T cells analyzed using flow cytometry. (E) Flow cytometric analysis of exhaustion marker expression (TIM-3 and LAG-3) in CD3^+^, CD4^+^ and CD8^+^ T cell populations following cytotoxic activity of LNP-transfected T cells normalized to electroporated T cells. (F) Mean percentage of CAR T cells expressing zero, one, two, or three exhaustion markers in electroporated T cells (upper) and LNP-transfected CD19-CAR T cells (lower) following cytotoxic activity. (G) Supernatants from migrated T cell killing assays were collected and analyzed for pro-inflammatory cytokines. Mean levels of granzyme B, IFN-γ, TNF-α, IL-8, and IL-10 are shown. Normalized to CD19-CAR frequency for each sample. Statistical analysis for differences between subpopulations was performed by Friedman test followed by Dunn’s multiple comparison test (C, D, and G) or paired *t* test (E). ∗ns, not significant; ∗*p* < 0.05, ∗∗*p* < 0.01, ∗∗∗*p* < 0.001, ∗∗∗∗*p* < 0.0001. Data represent mean ± SEM from *n* = 4 independent donors.

To assess whether reduced cellular stress from LNP-mediated mRNA delivery^28^^,^^47^ improves CAR T cell performance, we compared key functional attributes of CD19-CAR T cells generated by electroporation or LNP transfection. We evaluated CAR expression kinetics, migration capacity, cytotoxic activity, and exhaustion profiles following antigen-specific killing. T cells were first subjected to a transwell migration assay toward CCL21 over 3 h. Subsequently, live-cell imaging tracked cytotoxicity of migrated CD19-CAR T cells against NALM6 target cells (CD19^+^ GFP^+^) over 28 h, with measurements every 4 h (Figure 7A). Given their higher CAR density, electroporated cells were anticipated to show stronger killing. Surprisingly, LNP-transfected CAR T cells achieved comparable target cell killing (Figure 7B), despite reduced CAR frequency (Figure S7A). No killing was observed without CCL21, demonstrating that cytotoxicity was strictly dependent on prior migration (Figure S7B). Although the absolute cytotoxic capacity appeared comparable between groups (Figure 7B), adjusting for differences in CD19-CAR^+^ frequencies between LNP-transfected and electroporated CD19-CAR T cells revealed that LNP-transfected T cells trended toward enhanced per-cell cytotoxicity, as indicated by the area under the curve (AUC) of live target cell counts over time relative to untouched T cells (Figure 7C). This normalization accounts for differences in transfection efficiency between the two IVT mRNA delivery methods, enabling a direct comparison of the functional quality of individual CAR T cells. To explore this further, we analyzed chemotactic behavior and exhaustion status. LNP-transfected T cells trended to show improved migration toward CCL21 (Figure 7D), despite CCR7 expression levels being lower in LNP-transfected T cells compared to electroporated cells (Figure S7C), indicating higher migration potential and cell fitness. Phenotypic profiling after the killing assay revealed markedly lower expression of exhaustion markers TIM-3 and LAG-3 across CD3^+^, CD4^+^, and CD8^+^ subsets in the LNP group (Figures 7E and S7D), while PD-1 levels remained unaffected (Figure 7E). This profile was consistent with a more sustained, less exhausted functional state (Figure 7F). To better understand comparable killing efficacies observed despite reduced CAR expression in LNP-transfected CD19-CAR T cells, we analyzed cytokine secretion profiles of the killing assay supernatant. Electroporated CD19-CAR T cells exhibit enhanced pro-inflammatory cytokine secretion after 28 h of killing, including elevated IFN-γ, TNF-α and granzyme B levels (Figures 7G, S7E, and S7F). Additionally, electroporated cells secreted higher levels of IL-8, IL-10, MIP-1α (CCL3), and MIP-1β (CCL4) compared to LNP-transfected CD19-CAR T cells (Figures 7G, S7E, and S7F). To determine whether the enhanced cumulative cytotoxicity of LNP-transfected CD19-CAR T cells in the combined migration-killing assay was driven by improved migration or by superior intrinsic killing capacity, we assessed cytotoxic function independently of CCR7-mediated migration. CD19-CAR T cells were co-cultured with NALM6 and Raji target cells (both CD19^+^ GFP^+^) in different E:T ratios (1:3, 1:1, and 3:1) for a total observation period of 68 h and rechallenged with fresh tumor cells every 24 h (Figure S8A). As expected, untouched and mock-transfected T cells lacking CD19-CAR expression showed no cytotoxic activity. Both LNP-transfected and electroporated CD19-CAR T cells demonstrated comparable killing across both B cell lines and in all tested E:T ratios (Figure S8A). Cytometric analysis of T cells post-killing confirmed the higher exhaustion marker expression in electroporated cells (Figure S8B). Together, these data confirm that LNP-transfection of CAR T cells preserves intrinsic cytotoxic potency and limits exhaustion, independently of and in addition to superior migratory performance.

## Discussion

Our findings demonstrate that IVT mRNA provides a transient, non-integrating platform, which mitigates theoretical safety concerns by avoiding risks of insertional mutagenesis and irreversible genomic alterations associated with viral vectors or CRISPR-Cas9 systems,^11^^,^^12^^,^^13^^,^^14^^,^^18^ while enabling sophisticated multiplexing strategies and potentially enhancing functional efficacy.^28^^,^^29^^,^^30^^,^^31^^,^^32^^,^^45^^,^^65^ In certain non-malignant settings, such as autoimmune diseases, transient CAR expression may be sufficient to achieve durable clinical benefit,^10^^,^^66^^,^^67^ as suggested by recent observations in myasthenia gravis.^16^ Temporal control over CAR expression could facilitate precise dosing and redosing, directly addressing safety concerns linked to constitutive CAR expression.^28^^,^^44^ The absence of genomic integration and controlled expression kinetics align with regulatory priorities for safer cell engineering approaches, potentially easing clinical translation.^12^ Our multiplexing experiments illustrate the versatility of IVT mRNA for generating functional CAR T cells with additional capabilities. By co-delivering multiple transgenes with different subcellular localization and processing (CD19-CAR, CCR7, CXCR3, and GFP), we achieved functionally relevant co-expression levels of all encoded proteins addressing conventional limitations of DNA-based genome editing. As a model for functional enhancement, CCR7 co-expression with CD19-CAR promoted homing toward CCL21, thus potentially to secondary lymphoid organs and supported functional features associated with central-memory-like T cells linked to improved persistence and function.^36^^,^^37^^,^^52^ Importantly, CCR7 IVT mRNA rescued both migratory and cytotoxic function in CRISPR-generated CCR7 knock-out T cells to levels comparable with endogenously CCR7-expressing T cells. This approach highlights the possibility to compensate for the reduced central memory T cell abundance observed in elderly patients,^38^ thereby improving the robustness of autologous CAR T cell products in adoptive immunotherapy.

While electroporation resulted in approximately an order of magnitude higher CAR surface density than LNP-mediated mRNA delivery, transcriptomic, phenotypic, and functional analyses revealed advantages of LNP-mediated transfection, despite lower CAR expression levels. The frequently postulated “better cell fitness” of LNP-transfected T cells has typically been supported only by viability data,^45^ with limited mechanistic insight. Our study identifies and functionally validates multiple mechanisms through which LNP-mediated IVT mRNA delivery preserves functional fitness of CAR T cells.

Single-cell transcriptomic profiling of electroporated versus LNP-transfected cells suggested differences across various T cell function programs, including T cell activation and cytotoxic function. The identification of an electroporation-specific CD4^+^ T cell cluster (cluster 8) characterized by upregulation of cell stress response and DNA damage response genes (*TRIB3*, *DDIT3*, *CCPG1*, *SESN2*, *DNAJB9*, and *ATF3*)^55^^,^^56^^,^^62^^,^^63^^,^^64^ suggest that a substantial subset of electroporated cells undergo endoplasmic reticulum stress and activation of DNA damage response pathways, phenotypes linked to premature exhaustion, immunosenescence and dysfunction. Pathway enrichment analysis confirmed pronounced endoplasmic reticulum stress, unfolded protein response, and consistent with disturbed proteostasis in cluster 8, which may contribute to elevated annexin V staining and reduced functional capacity of electroporated CD19-CAR T cells. In contrast, the LNP-transfected CD19-CAR T cells displayed signatures of physiological innate immune sensing of LNPs, which were not associated with stress pathway activation at transcriptome or phenotypic levels. In line with this, higher annexin V staining without accompanying cell death was observed in electroporated compared to LNP-treated T cells and occurred regardless of whether mRNA was present, indicating transient cell stress induced by electroporation itself.

Functionally, higher CAR molecule density did not translate into superior CAR T cell performance, as LNP-transfected cells exhibited comparable cytotoxicity across various human *in vitro* potency assays. This apparent paradox indicates that supraphysiologic CAR expression levels may not proportionally enhance cytotoxic output and could instead promote activation-induced dysfunction or early exhaustion. In contrast, the lower but more controlled CAR expression observed following LNP-mediated IVT mRNA delivery may preserve signaling balance and functional fitness at the single-cell level, resulting in comparable or even enhanced cumulative cytotoxicity despite reduced overall transfection efficiency. Functional analysis demonstrated enhanced per-cell cytotoxic activity in LNP-transfected cells, reduced expression of exhaustion markers (TIM-3 and LAG-3), robust activation indicating preserved functional fitness,^68^ higher migratory capacity, and a controlled cytokine profile compared to electroporated T cells. Electroporated T cells showed elevated pro-inflammatory cytokines (IFN-γ, TNF-α, granzyme B, IL-8, IL-10, MIP-1α, and MIP-1β), several of which are linked to impaired T cell function and immunosenescence. IL-10, despite anti-inflammatory properties, can suppress CD8^+^ T cell activity and contribute to exhaustion,^69^^,^^70^ whereas elevated IL-8 has been associated with poor prognosis and reduced benefit from immune checkpoint blockade.^71^^,^^72^ Both IL-8 and IL-10 are involved in the pathogenesis of cytokine release syndrome.^73^ The controlled cytokine milieu in LNP-transfected cells suggests reduced inflammatory burden, potentially sustaining therapeutic function and improving safety. These phenotypic and functional differences reflect the distinct transcriptomic cell stress profiles induced by the two delivery methods. Endocytic uptake via LNPs may provide a more physiological processing route compared to the membrane permeabilization of electroporation, which can trigger stress responses and background activation. This gentler delivery mode preserves cellular integrity and limits non-specific activation, factors that together may enhance both safety and functional durability. On the one hand, differences across electroporation platforms may result in distinct CAR T cell product characteristics, making a complete generalization of electroporation-induced cell phenotypes practically impossible. On the other hand, in this study electroporation was extensively preoptimized using the Lonza 4D-Nucleofector to achieve optimal viability and transfection efficacy, before being compared to LNP transfection. Nonetheless, even highly optimized electroporation was associated with alterations in several T cell functional parameters, whereas LNP transfection did not. Both transfection methods preserved early differentiated T cell subsets (predominantly T_naive like_ and T_CM_ cells) associated with superior functional capacity and metabolic fitness in adoptive T cell therapy.^36^^,^^37^ We observed higher transgene expression in T_naive like_ and T_EMRA_ subsets compared to effector memory cells, likely reflecting metabolic differences between differentiation stages.^74^ Our results suggest that delivery platforms which prioritize functional durability, rather than absolute CAR abundance, may ultimately be more advantageous for clinical efficacy. Although our results were obtained from a single LNP formulation, they demonstrate the potential of nanocarriers for achieving improved functional profiles and provide a broadly applicable blueprint for functional cell characterization, which may enable more meaningful preclinical evaluation of nanocarriers and transfection methods in general.

Electroporation remains widely used for *ex vivo* CAR T cell engineering^39^^,^^40^^,^^41^^,^^42^ but poses hurdles for broad clinical adoption, including the need for leukapheresis, extensive cell manipulation, expansion, and reinfusion.^75^ This process is time-consuming, costly, and highly dependent on the specific electroporation hardware. LNP-mediated mRNA delivery offers several practical advantages in the *ex vivo* setting, including reduced cellular stress, preserved functional fitness,^28^^,^^44^^,^^45^^,^^46^^,^^47^ single-step multiplexing capability, and scalability. These features could streamline manufacturing and improve the CAR T cell product quality. LNPs are clinically advanced nanocarriers forming the basis of approved therapeutics.^76^^,^^77^ Furthermore, LNPs are being explored for *in vivo* CAR T cell generation, which would eliminate the need for any *ex vivo* cell manipulation, potentially reducing manufacturing time and increasing accessibility by delivering health-economic benefits.^31^^,^^44^^,^^51^ Although our study focused exclusively on *ex vivo* delivery, the functional advantages observed here (reduced exhaustion, preserved migratory potential, and controlled activation) provide a strong rationale for further exploration in the *in vivo* CAR setting.^31^ Direct assessment of persistence and long-term function in advanced animal disease models will be essential to determine to what extent these functional advantages will translate to therapeutic efficacy in patients. For autoimmune diseases in particular, mRNA-based CAR T cell engineering aligns with the concept of an “immune reset”, in which transient CAR activity eliminates pathogenic B cells without requiring long-term persistence.^24^^,^^25^ The reduced exhaustion phenotype of LNP-transfected T cells may preserve function in inflammatory environments, while multiplexing approaches, such as incorporating trafficking modulators like CCR7, enable more precise action.

In summary, this study establishes IVT mRNA multiplexing as a versatile approach for equipping CAR T cells with additional functional traits and provides mechanistic evidence on how delivery method influences phenotype and performance. While electroporation’s efficacy for high-level transgene expression is constrained by trade-offs in cell functionality, LNP-mediated delivery offers distinct functional advantages, supporting development of safer, more adaptable, and potentially more accessible cell and gene therapeutics.

## Materials and methods

### Blood sample processing

PBMCs were isolated from heparinized whole blood samples collected from healthy donors. All donors provided written informed consent, and the study was approved by the Ethics Committee of Charité – Universitätsmedizin Berlin (approval no. EA4/091/19).

### T cell isolation and polyclonal activation

PBMCs were isolated from healthy donors using Biocoll (Biochrom) gradient centrifugation, and CD3^+^ T cells were enriched using magnetic cell separation with CD3 Microbeads (Miltenyi Biotec). T cells were activated using plate-bound anti-CD3 (1 μg/mL, Okt3, eBioscience) and anti-CD28 (1 μg/mL, BioLegend) antibodies for 48 h. Primary human T cells were cultured in a humidified incubator at 37°C in 5% CO_2_ using CTL medium consisting of a 1:1 mixture of advanced RPMI (Gibco) and Click’s medium (Fujifilm) supplemented with 10% fetal calf serum (FCS, PAA), 1% GlutaMAX (Gibco), and rhIL-7 and rhIL-15 (both 10 ng/mL, CellGenix) or ImmunoCult-XF T cell Expansion Medium (Cell signaling Technology) supplemented with 10% FCS (PAA) and rhIL-7 and rhIL-15 (both 10 ng/mL, CellGenix).

### CRISPR-Cas9-mediated CCR7 knock-out

On day 3 after T cell isolation, 1 × 10^6^ T cells were prepared for CRISPR-Cas9 editing by resuspension in P3 electroporation buffer from the 4-D Nucleofector X Kit S (Lonza). Riboprotein complexes (RNPs) were assembled by combining 2 single-guide RNAs (sgRNAs; sgRNA1: GGGGTCACGGACGATTACAT and sgRNA2: GGGCAGGTAGGTATCGGTCA), poly(L-glutamic acid) (100 μg/μL, Sigma) and recombinant Alt-R HiFi Streptococcus pyogenes Cas9 protein V3 (Integrated DNA Technologies) in a 0.96:1:0.8 volume ratio. T cells were electroporated with the RNP complex using the Amaxa Nucleofector 4D system (Lonza) with program EO-115. Following electroporation, T cells were immediately recovered in pre-warmed CTL medium and incubate at 37°C for 10 min before transferring to a 48-well plate for further cultivation. Following CRISPR-Cas9 electroporation, CCR7 knock-out T cells were recovered and transfected with IVT mRNA (CD19-CAR ± CCR7) 3 days later.

### *In vitro* transcription

The plasmid vector template pRNA2-(A) 128 (Addgene plasmid #174 006) was used as a backbone, containing a 5*′*-UTR, inserted coding sequence, human β-globin 3*′*-UTR, upstream T7 promotor (upstream), and a 128-long polyadenine (poly(A)) tail at 3*′* end. Insert sequences encoding CCR7, CD19-CAR, CXCR3, or EGFP (Table S1) were designed *in silico* and synthesized by Integrated DNA Technologies. The sequences for CD19-CAR and CXCR3 were cloned into the plasmid vector using specific primers (Table S2) (Integrated DNA Technologies) and In-Fusion Cloning (Takara) according to the manufacturer’s protocol. The sequence for CCR7 was cloned into the plasmid vector using the restriction enzymes *Not I* und *Hind III* (New England Biolabs). The DNA fragment spanning from the T7 promotor to the poly(A) tail, including the coding sequence, was PCR amplified using specific primers binding at the T7 promotor and the poly(A) tail (Table S2) and KAPA High Fidelity DNA Polymerase (Roche). The PCR product was purified using DNA Clean & Concentrator-5 kit (Zymo Research) and served as a template for IVT.

*In vitro* transcription was performed using the TranscriptAid T7 High Yield Transcription Kit (Thermo Fisher Scientific). The reaction mixture contained the purified DNA template, nucleotide trisphosphate (ATP, GTP, and CTP at 100 nM each), anti-reverse cap analog (ARCA; 100 nM; Jena Bioscience) and the provided enzyme mix and reaction buffer. For UTP, the chemically modified nucleotide-5-methoxy-UTP (5moU; Jena Bioscience) was used at 100 nM concentration. The transcription reaction was incubated for 2 h at 37°C. To remove template DNA, DNase I was added to the IVT reaction and incubated for 15 min at 37°C. mRNA was purified using lithium chloride (LiCl) precipitation by adding distilled water and LiCl to the reaction and incubating at −20°C overnight. Following centrifugation, the pellet was washed with 70% ethanol (VWR), air-dried, and resuspended in RNase-free water. mRNA integrity was assessed by electrophoresis on a 1.2% RNase-free agarose gel and mRNA concentration was determined using UV-visible spectroscopy (NanoDrop One spectrophotometer, Thermo Fisher Scientific).

### mRNA delivery using electroporation

On day 3 after T cell isolation or CRISPR-Cas9-mediated CCR7 knock-out, polyclonally activated T cells were harvested following polyclonal stimulation and either cultured for an additional day without stimulus or directly used for mRNA transfection depending on the experimental setup. For electroporation, 1 × 10^6^ T cells were resuspended in P3 electroporation buffer from the 4-D Nucleofector X Kit S (Lonza) and electroporated using the Amaxa Nucleofector 4D system (Lonza). Unless otherwise specified, program EO-115 was used, alternative programs tested included DN100, EH100, EH115, EO210, and FI115. IVT mRNA was added at 1 μg unless indicated otherwise (2 μg and 3 μg were also tested) per 1 × 10^6^ T cells, using either single IVT mRNA constructs or multiplexed mRNA combinations. Following electroporation, T cells were immediately recovered in pre-warmed CTL medium and incubated at 37°C for 10 min before transferred to a 48-well plate for further cultivation. Electroporated T cells without mRNA served as mock-transfected control. Electroporated T cells were used for functional assays 4 h post-transfection.

### IVT mRNA delivery using lipid nanoparticles

IVT mRNA was encapsulated into lipid nanoparticles using the GenVoy-ILM T-cell kit (Precision NanoSystems). For formulation, 11 μg of mRNA was diluted with RNase-free water and 10× formulation buffer in a total volume of 35.2 μL. The dilution buffer, mRNA working solution, and lipid mix were microfluidic mixed using the NanoAssemblr Spark device with setting 3. The formulated mRNA-LNPs were diluted 1:1 with dilution buffer to achieve a final concentration of approximately 30 ng/μL.

Polyclonally activated T cells were harvested on day 3 after T cells isolation and 1 × 10^6^ T cells were transferred into a 48-well plate in either ImmunoCult media or CTL medium without FCS. 1 μg of formulated mRNA-LNP complex and 4 μg/mL ApoE (Precision NanoSystems) or only ApoE were added to the cells. After 4 h of incubation, 10% FCS was added to each well. Cells treated with ApoE alone without mRNA-LNP complex served as mock-transfected controls and cells under FCS withdrawal without ApoE and mRNA-LNP complex served as untouched controls. LNP-transfected T cells were used for functional assays 20 h post-transfection.

### T cell migration and target killing

mRNA-transfected T cells were counted and 0.4 × 10^6^ T cells were seeded into the upper chamber of a 96-well transwell plate (HTS Transwell 96-well, 3.0 μm Pore, Dow Corning). RPMI medium with 10% FCS containing recombinant CCL21 (200 ng/mL; R&D Systems) was placed in the lower chamber of the transwell system. RPMI medium without CCL21 served as a negative control. T cells were allowed to migrate toward the lower chamber for 3 h in a humidified incubator at 37°C with 5% CO_2_. After the 3-h migration period, migrated cells were collected co-cultured with 3 × 10^4^ CD19-expressing GFP-labeled NALM6 cells on a fibronectin-coated 96-well clear bottom imaging plate (PhenoPlate, PerkinElmer). The total GFP-positive area was measured every 4 h over a 28-h period using the ImageXpress PICO automated imaging system (Molecular Devices) to assess target cell killing.

Following migration and target cell killing, the chemotactic index (CI) was calculated as the ratio of the absolute number of cells migrating toward CCL21 to the absolute number of cells migrating toward the control medium without CCL21. The absolute number of migrated T cells and exhaustion marker expression was determined using a Cytoflex LX flow cytometry (Beckman Coulter).

### Serial killing assay

To analyze the serial killing capacity of the CD19-CAR T cells, mRNA transfected T cells were co-cultured with either NALM6 or Raji tumor cells (both CD19^+^ GFP^+^) at defined E:T ratios (1:3, 1:1, and 3:1) on a fibronectin-coated 96-well clear bottom imaging plate (PhenoPlate, PerkinElmer). Fresh tumor cells were added every 24 h at distinct E:T ratios over a total period of 68 h based on the initial CAR T cell number seeded. The total GFP-positive area was quantified every 4 h using an ImageXpress PICO automated imaging system (Molecular Devices) to monitor target cell killing. The expression of exhaustion markers was assessed at the end of the killing assay by flow cytometry using a Cytoflex LX flow cytometry (Beckman Coulter).

### Flow cytometric analysis

All flow cytometric analyses were performed using the Cytoflex LX flow cytometry (Beckman Coulter) and analyzed with FlowJo-10 software (Tree Star). T cells were stained with fluorophore-conjugated antibodies (all from BioLegend, unless stated otherwise). LIVE/DEAD Fixable Blue Dead Cell Stain (Invitrogen) was used to exclude dead cells in all analyses.

#### mRNA transfection efficacy

To assess mRNA transfection efficacy in primary human T cells, CD19-CAR detection was performed using either PE-labeled human CD19 protein, His tag (Acrobiosystem) or a fluorophore-conjugated anti-Myc antibody (9B11; Cell signaling Technology) to detect the Myc-tag present in the CAR construct. Cells were stained with extracellular markers including flurophore-conjugated antibodies against human CD3 (OKT3), CD4 (SK3), CD8 (RPA-T8), CD45RA (HI100), and CCR7 (G043H7). For multiplexed mRNA-transfection analysis, GFP signal was measured, and cells were stained with fluorophore-conjugated antibodies against Myc (9B11), CCR7 (G043H7), and CXCR3 (G025H7).

#### Antigen-specific stimulation of CD19-CAR T cells

To assess activation and cytokine production of CD19-CAR T cells following antigen encounter, CD19-expressing NALM6 cells were co-cultured with CD19-CAR T cells at a 3:1 E:T ratio for 16 h. To capture intracellular cytokine production, 2 μg/mL Brefeldin A (Sigma-Aldrich) was added after the first hour of stimulation. T cells were stained using fluorophore-conjugated antibodies against human CD3 (OKT3), CD4 (SK3), CD8 (RPA-T8), CD45RA (HI100), CCR7 (G043H7), IFN-γ (4S.B3), TNF-α (MAb11), IL-2 (MQ1-17H12), CD137 (4B4-1), and CD154 (24–31). CD19-CAR T cells without NALM6 cell stimulation served as negative controls.

#### Exhaustion marker expression

Following migration and target-cell killing assays, exhaustion marker expression profiles were assessed on T cells. Cells were stained using fluorophore-conjugated antibodies against human CD3 (OKT3), CD4 (SK3), CD8 (RPA-T8), LAG-3 (3DS223H, Invitrogen), PD-1 (EH12.2H7), and TIM-3 (F38-2E2).

### Cytokine analysis

Supernatants from migrated T cells following CCL21-directed migration and 28 h of target cell killing were collected and analyzed for cytokine production. Samples were measured at two dilutions (1:2 and 1:10) using the Human Immunotherapy Luminex Performance Assay 25-plex Fixed Panel (R&D Systems) according to the manufacturer’s protocol.

### scRNA-seq sample and library preparation

IVT mRNA transfected T cells were generated as described previously and processed 24 h post-transfection. To enable multiplexing of donors and experimental conditions, each sample was labeled with a unique TotalSeq-C hashtag antibody (BioLegend). Briefly, cells were incubated with 1 μg of the respective hashtag antibody in 50 μL of staining buffer (DPBS supplemented with 0.5% BSA and 2 mM EDTA) for 30 min at 4°C. After incubation, cells were washed 3 times with a total of 4.5 mL loading buffer (DPBS +1% BSA). All conditions were subsequently counted using a CytoFlex flow cytometer and pooled in equal proportions. The pooled cell suspension was passed through a 40 μm cell strainer (FlowMi, Merck) to remove aggregates. For each reaction, 50,000 cells were loaded into the Chromium iX Controller (10× Genomics), following the manufacturer’s instructions for the 10× Genomics 5′ v.2 kit. Reverse transcription, cDNA amplification, and library construction were performed according to the manufacturer’s instructions. Library quantification was carried out using a Qubit fluorometric assay (Thermo Fisher Scientific), and quality control was assessed using the Agilent TapeStation 4150. Pooled libraries were quantified with a KAPA qPCR assay prior to sequencing.

#### Sequencing and data processing

Sequencing was performed on an Illumina NovaSeq X system using three lanes of a 10B flow cell in paired-end mode with 100 cycles. Target sequencing depths were 40,000 reads per cell for gene expression libraries and 5,000 reads per cell for antibody capture (hashtag) libraries. Raw base call files were demultiplexed and processed using cellranger (v 9.0.1, 10× Genomics) aligning reads to the GRCh38 reference genome. Donor genotype assignment was performed with cellSNP-lite (v 1.2.2) using common SNPs from phase 3 of the 1000 Genomes Project. Donor demultiplexing was performed using vireoSNP (v 0.5.9). Cells identified as doublets or unassigned were excluded from further analyses.

#### Data demultiplexing and quality control

Downstream analysis was performed in R using Seurat (v 5.4.0). Hashtag-oligo (HTO) demultiplexing was carried out with the Seurat HTODemux function using default parameters. Quality control was assessed by evaluating ridge and t-SNE plots, and cells labeled as doublets or negative were removed. For gene expression (GEX) data, the number of detected genes, number of unique molecular identifiers (UMIs), and the percentage of mitochondrial transcripts were normalized per library by dividing each value by the median. Cells with normalized values <0.5 for detected genes and UMIs, and >2 for percent mitochondrial transcripts, were excluded to remove low-quality cells. Cluster showing high percent mitochondrial transcripts and low number of detected genes and UMIs were additionally removed.

#### Data integration, clustering, and annotation

Filtered datasets were integrated iteratively using Seurat’s IntegrateLayers function (method = CCAIntegration). Clustering was performed using Seurat’s shared nearest neighbor (SNN) graph-based method combined with the Leiden algorithm. Clusters were annotated based on canonical T cell and PBMC marker expression. MAIT cell clusters were excluded, retaining only CD4^+^ and CD8^+^ T cells. The resulting T cell dataset was integrated across donors for downstream analyses.

#### Differential expression testing

Differential expression analysis was performed using a paired pseudobulk approach at the sample level. For each cell type, transfection method and donor, raw UMI counts were summed across cells to obtain a sample-level count matrix, which was analyzed with DESeq2 (v 1.46.0). Differential expression across all transfection groups was first evaluated using a likelihood ratio test (LRT) comparing a full model including the transfection method to a reduced model lacking this term, to identify genes with significant overall regulation. Log2 fold change and adjusted *p* values for specific pairwise contrasts between groups were then obtained using the Wald test within the same DESeq2 framework. To obtain stable effect size estimates, log fold-change shrinkage was applied using the ashr package,^78^ and shrunken Log2FoldChange was reported throughout. Genes with reported NA values for adjusted *p* values were not interpreted and excluded from further analysis and visualizations.

#### Gene ontology enrichment and module score analyses

GO term enrichment analysis for DEGs from cluster 8 was performed using the clusterProfiler R package (v 4.14.0). The top 50 upregulated DEGs were selected based on log2 fold change values (adjusted *p* < 0.05, baseMean >50). Enrichment analysis employed the enrichGO function with org.Hs.e.g.,.db (v 3.20.0) as the annotation database, “ENTREZID” as key type, and “BP” as ontology. Gene ontology-based module scores were calculated using curated GO term gene sets retrieved via AnnotationDbi (v 1.68.0). Per-cell module scores were computed using Seurat’s AddModuleScore function with default parameters. Statistical comparisons of median module scores across groups were performed using the stats (v 4.4.3) Kruskal-Wallis test, followed by rstatix (v 0.7.3) Dunn’s post hoc test with Holm adjustment for multiple testing. The same approach was applied to assess differences in cluster frequencies across transfection methods.

### Data visualization

Differential expression results were visualized using the EnhancedVolcano R package (v 1.24.0). Additional plots were generated with ggplot2 (v 4.0.1). Data wrangling and reshaping were performed using tidyr (v 1.3.2) and dplyr (v 1.1.4).

## Statistics

GraphPad Prism 10 (GraphPad Software) was used to generate graphs and perform statistical analysis of the data. To test for normal Gaussian distribution, the Kolmogorov-Smirnov test was performed. For comparison of 2 normally distributed datasets, a paired *t* test was conducted. Multiple group comparisons were analyzed using one-way ANOVA with Tukey’s post hoc test or using Friedman test followed by Dunn’s multiple comparison. For correlation analysis, Pearson correlation coefficients were calculated, and regression lines were generated based on Spearman’s rank correlation using the robust regression function provided by GraphPad Prism. Statistical analysis for differences between subpopulations was performed using two-way repeated measures ANOVA with Šidák’s multiple comparison test. Statistical significance is indicated as follow: ∗ns, not significant; ∗*p* < 0.05; ∗∗*p* < 0.01; ∗∗∗*p* < 0.001.

## Data and code availability

All data needed to evaluate the conclusions in the paper are present in the paper and/or the supplemental information. There are no restrictions on the use of materials. All data generated in this study are available as source data or from the corresponding author on reasonable request.

## Acknowledgments

We thank all voluntary blood donors for their generous contributions. We thank Dr. Dimitrios L. Wagner for kindly providing the Raji target cell line (CD19^+^ GFP^+^) used in this study. We acknowledge helpful support by Benedikt Obermayer and the Core Unit Bioinformatics at the BIH. This study was supported by the German Federal Ministry of Education and Research (BMBF) through the BIH Center for Regenerative Therapies (to M.S.-H.) and the CONAN project (16GW0328K to M.S.-H.). It also received funding from the ERDERA consortium (European Rare Diseases Research Alliance) under the European Union’s Horizon Europe research and innovation program, grant agreement no. 101156595 and from the Stiftung Charité through a BIH_PRO_677 Visiting Professor Grant (to C.P.). The funders had no role in study design, data collection and analysis, decision to publish, or preparation of the manuscript.

## Author contributions

S.P. planned and performed experiments, analyzed the results, composed the figures, interpreted the data, and wrote the manuscript. M.F.-S. and S.S. contributed to establishing the methodology and to data interpretation. A.L.H. and S.B. performed single-cell library preparation, sequencing, data processing, and bioinformatic analyses and contributed to data interpretation and visualization. A.M.K., R.F., and C.S. performed experiments with support from L.H. D.N.S., G.K., and C.P. L.E.S. provided overall conceptual guidance and expert input to data interpretation. H.-D.V. and M.G. provided essential infrastructure and oversight of data interpretation. M.S.-H. and N.M.D. led the project, conceptualized the study, designed the research, interpreted the data, and wrote the manuscript. All authors discussed, commented on, and approved the final version.

## Declaration of interests

All authors declare no competing interests.

## Declaration of generative AI and AI-assisted technologies in the writing process

During the preparation of this work the author(s) used Claude by Anthropic (claude.ai) in order to restructure and refine sentences and paragraphs for clarity and flow. After using this tool/service, the authors reviewed and edited the content as needed and take full responsibility for the content of the publication.

## Supplemental information

Document S1. Figures S1–S8 and Tables S1 and S2

Document S2. Article plus supplemental information
