# Supplementary material for: Lipid nanoparticle mRNA delivery preserves CAR T cell cytotoxicity and limits exhaustion compared to electroporation
Source: Mol Ther Nucleic Acids. 2026 Apr 28;37(2):102929. doi: 10.1016/j.omtn.2026.102929 (PMC13139981; doi:10.1016/j.omtn.2026.102929)
Supplement: Document S1. Figures S1–S8 and Tables S1 and S2 [file mmc1.pdf]

OMTN, Volume *37*

Supplemental information

Lipid nanoparticle mRNA delivery preserves CAR T cell cytotoxicity and limits exhaustion compared to electroporation

Samira Picht, Martí Farrera-Sal, Anna L. Hiller, Sophia Brumhard, Antonia M. Klaas, Sarah Schulenberg, Rebecca Friedrich, Cedric Scholz, Lisa Hemmerling, David N. Simon, Gerhard Krönke, Chantal Pichon, Leif E. Sander, Hans-Dieter Volk, Manfred Gossen, Michael Schmueck-Henneresse, and Norman M. Drzeniek

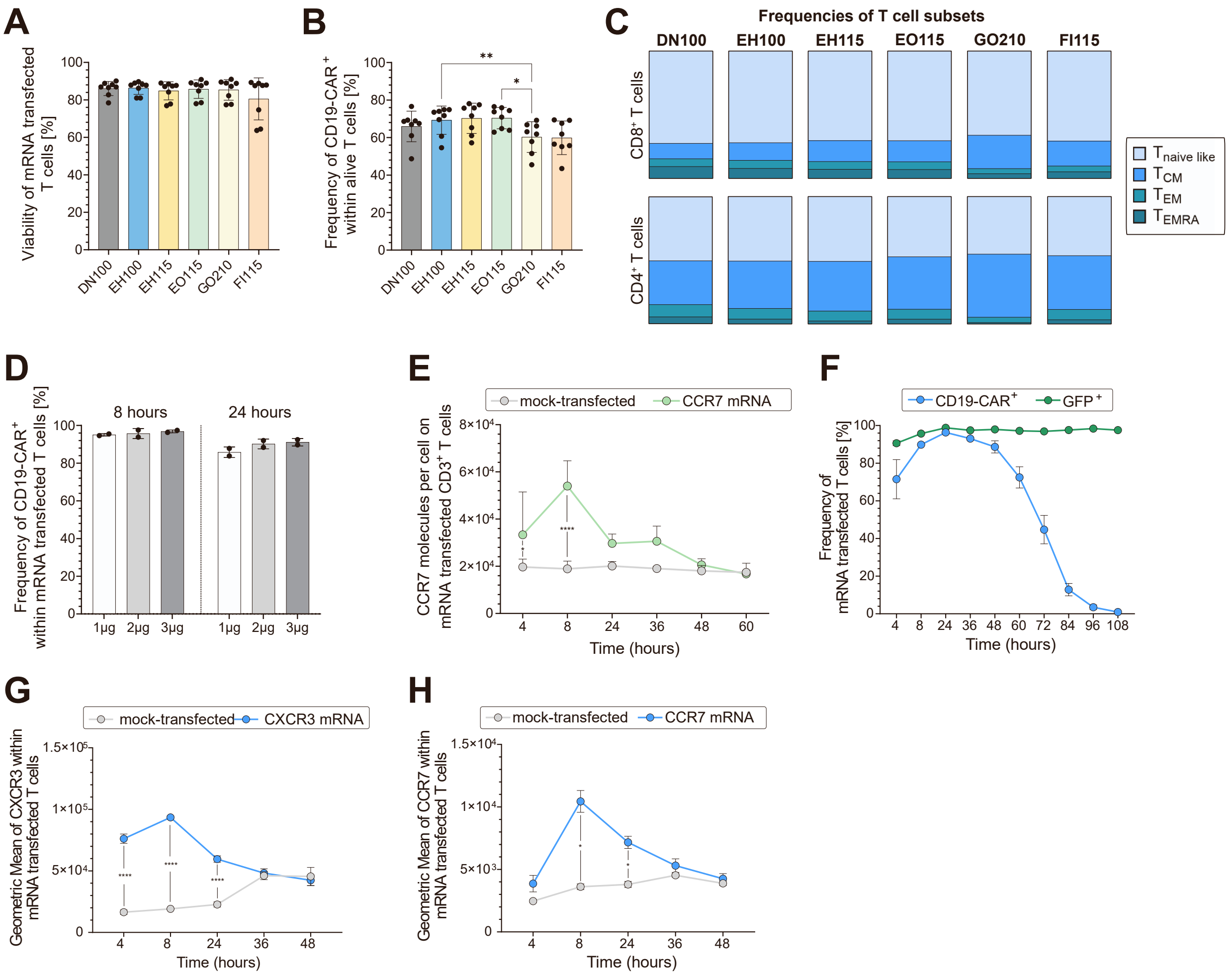

Figure S1: Optimization of electroporation conditions and characterization of multiple mRNA transfection in polyclonally activated T cells. (**A, B, C**) Viability (**A**), frequency of CD19-CAR⁺ (**B**) and memory phenotype characteristic of CD19-CAR⁺ T cells within CD8⁺ (upper panel) and CD4⁺ (lower panel) populations (**C**) in mRNA transfected T cells using different electroporation programs (DN100, EH100, EH115, EO115, EO210, FI115) of the Lonza 4D-Nucleofector system 24 hours post-transfection. Data represent mean ± SD from n=8 independent donors. (**D**) Frequency of CD19-CAR⁺ T cells 8- and 24-hours post-transfection using 1 µg, 2 µg and 3 µg of CD19-CAR mRNA via electroporation. Data represent mean ± SD from n=2 independent donors. (**E**) CCR7 expression levels in co-transfected T cells over 60 hours post-transfection compared to mock-transfected T cells. (**F**) Frequency of CD19-CAR⁺ T cells and GFP⁺ T cells within multiplexed CD3⁺ bulk T cells. (**G, H**) Expression levels of CXCR3 (**G**) and CCR7 (**H**) in multiplexed CD3⁺ bulk T cells compared to mock-transfected controls. Statistical analysis for differences between subpopulations was performed by two-way repeated measures ANOVA with Šidák's multiple comparison test or by one-way ANOVA with Tukey's post hoc test. *ns: not significant, *p<0.05, **p<0.01, ***p<0.001. Data represent mean ± SEM from n=4 independent donors.

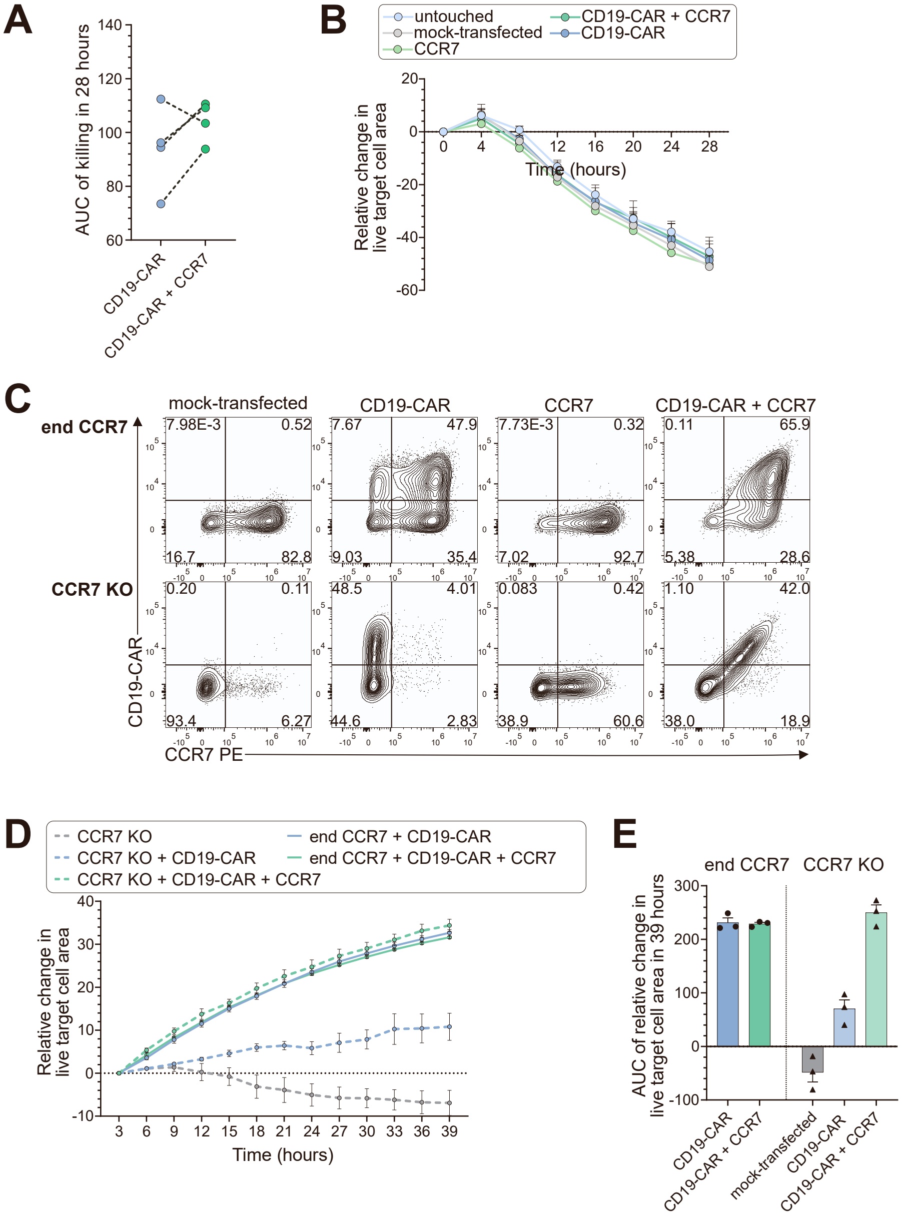

Figure S2: Functional characterization of CCR7 and CD19-CAR co-transfected T cells in migration and cytotoxicity assays. (**A**) Area under the curve (AUC) analysis of total killing capacity over the 28 hours observation period for CD19-CAR-only transfected T cells and CD19-CAR/CCR7 co-transfected T cells highlighting inter-donor variability. (**B**) Killing efficacy of CD19-CAR T cells after spontaneous migration. Cytotoxic efficacy against NALM6 (CD19⁺ GFP⁺) target cells by untouched T cells, mock-transfected T cells, CD19-CAR-only transfected T cells, CCR7-only transfected T cells and CD19-CAR/CCR7 co-transfected T cells. Killing was assessed over 28 hours using live-cell imaging every 4 hours following spontaneous T cell migration toward medium without additives. Data represent mean ± SD from n=4 independent donors. (**C**) Gating strategy for analyzing the transfection efficacy of CD19-CAR-only transfected T cells, CCR7-only transfected T cells and CD19-CAR/CCR7 co-transfected T cells comparing endogenous CCR7 expressing (end CCR7; upper panel) and CCR7 knock-out (CCR7 KO; lower panel) T cells. Representative flow cytometry plots showing expression of CCR7 and CD19-CAR in CD3⁺ T cells at 4 hours post-transfection. (**D**) Killing kinetics after CCL21-mediated migration. Time-course analysis of cytotoxicity against NALM6 (CD19⁺ GFP⁺) target cells by end CCR7 and CCR7 KO T cells transfected with IVT mRNA encoding for CCR7 and/or CD19-CAR over 39 hours. (**E**) Area under the curve (AUC) analysis of total killing capacity over the 39-hour observation period for the same groups. Data represent mean ± SD from n=3 independent donors, unless stated otherwise.

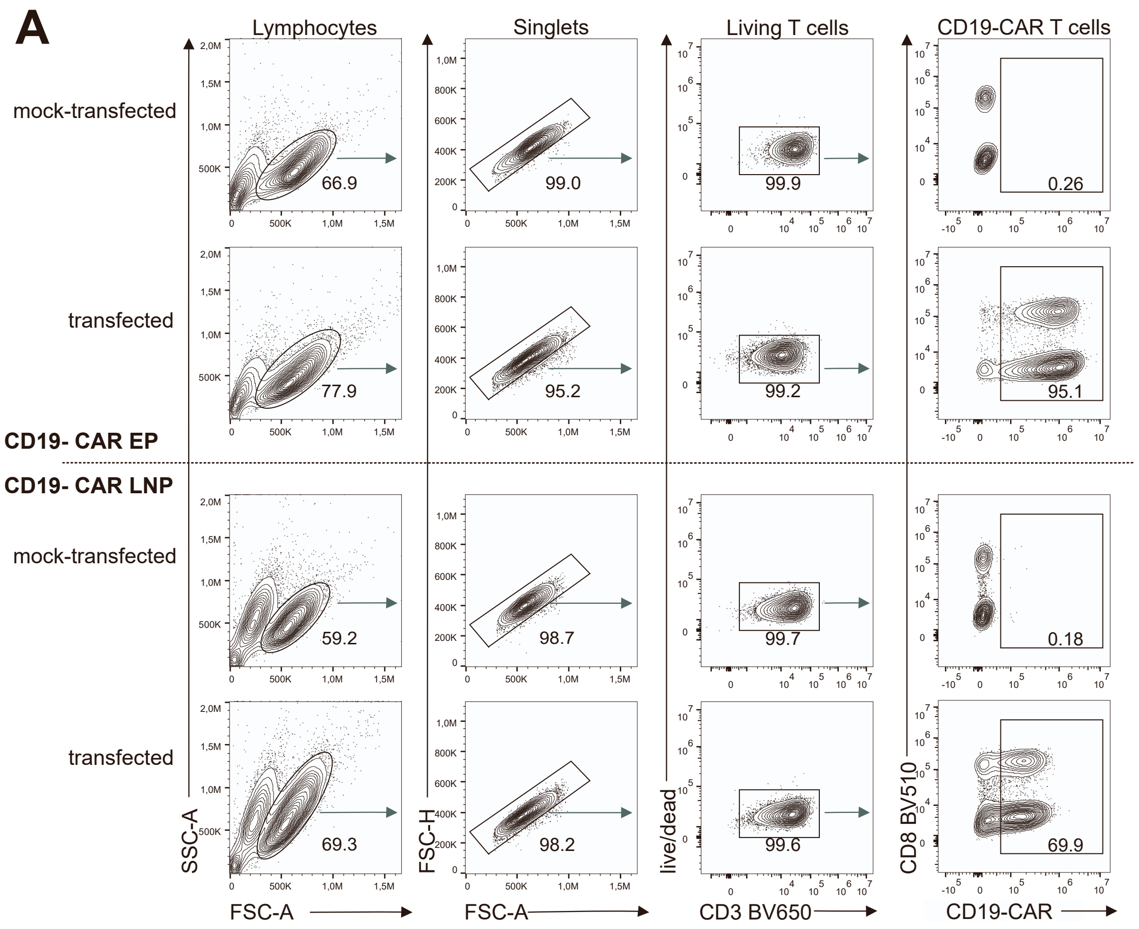

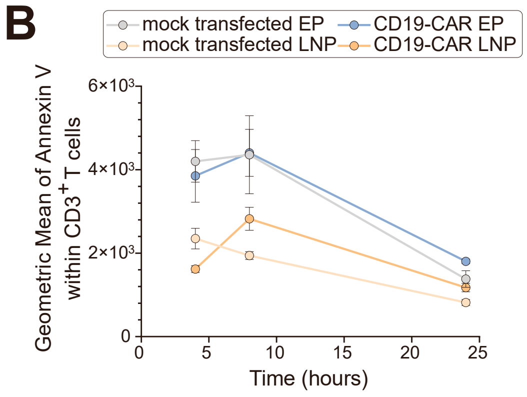

Geometric Mean of Annexin V

	mock-transfected EP			CD19-CAR EP		mock-transfected LNP
Time (h)	CD19-CAR EP	mock-transfected LNP	CD19-CAR LNP	mock-transfected LNP	CD19-CAR LNP	CD19-CAR LNP
4	ns	*	***	ns	**	ns
8	ns	**	ns	**	ns	ns
24	ns	ns	ns	ns	ns	ns

Figure S3: Comparison of electroporation and LNP-mediated mRNA delivery for CD19-CAR T cell engineering. (**A**) Gating strategy for analyzing transfection efficacy of CD19-CAR T cells comparing electroporation and LNP-transfection as IVT mRNA delivery methods**.** Representative flow cytometry plots showing sequential gating on lymphocytes, single cells, live $CD3^+$ T cells, and CD19-CAR expression in mock-transfected and mRNA-transfected T cells at 24 hours post-transfection for electroporation (EP; upper panels) and LNP delivery (LNP; lower panels). (**B**) Flow cytometric analysis of geometric mean of Annexin V in mock-transfected and mRNA-transfected T cells (EP or LNP) over 24 hours comparing electroporation and LNP-transfection as IVT mRNA delivery methods. Statistical analysis for differences between subpopulations was performed by two-way repeated measures ANOVA with Šidák's multiple comparison test. *ns: not significant, *p<0.05, **p<0.01, ***p<0.001. Data represent mean ± SD from n=5 independent donors, unless stated otherwise.

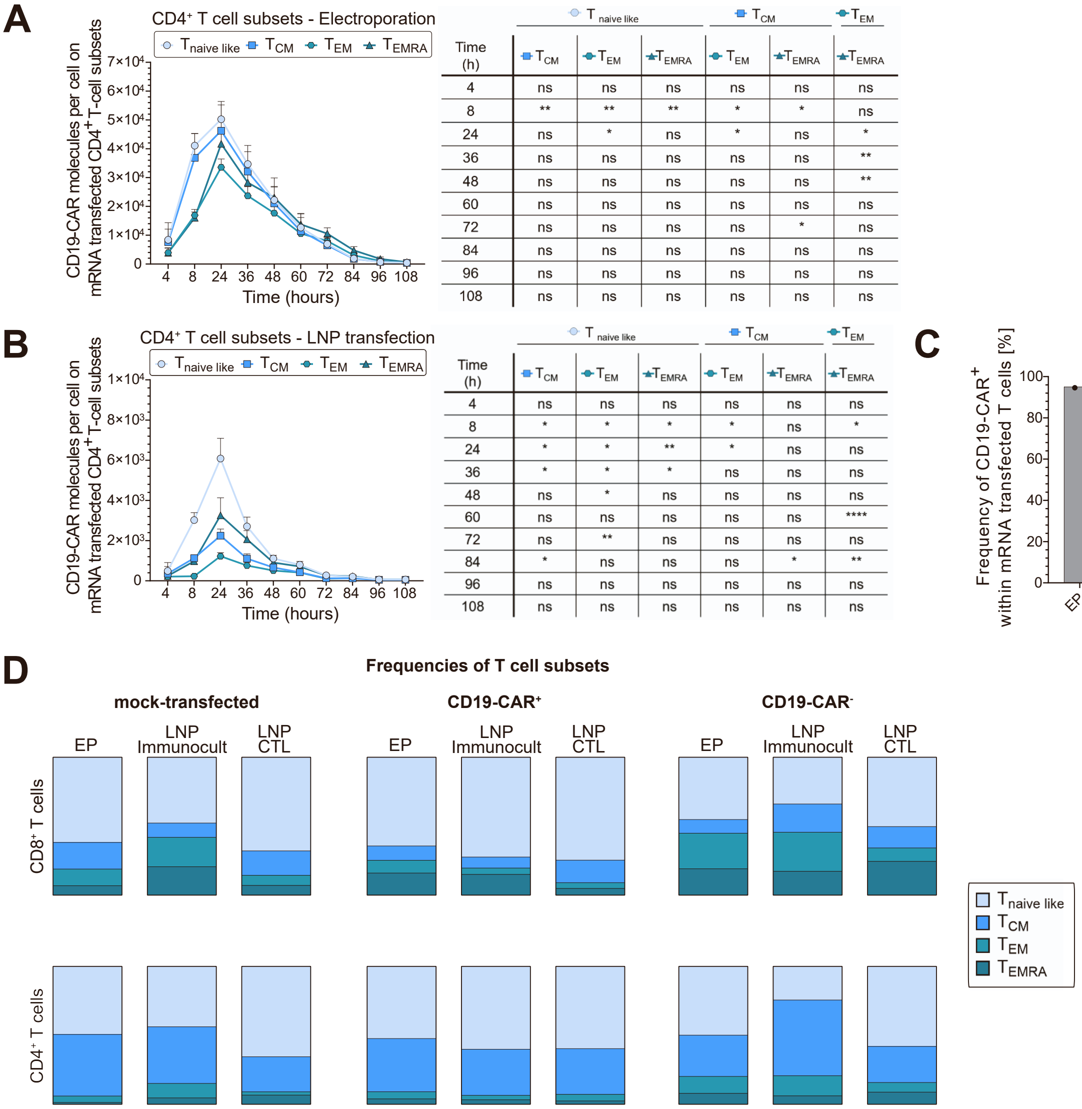

A — CD4⁺ T cell subsets - Electroporation

	$T_{naive\ like}$			T_{CM}		T_{EM}
Time (h)	T_{CM}	T_{EM}	T_{EMRA}	T_{EM}	T_{EMRA}	T_{EMRA}
4	ns	ns	ns	ns	ns	ns
8	**	**	**	*	*	ns
24	ns	*	ns	*	ns	*
36	ns	ns	ns	ns	ns	**
48	ns	ns	ns	ns	ns	**
60	ns	ns	ns	ns	ns	ns
72	ns	ns	ns	ns	*	ns
84	ns	ns	ns	ns	ns	ns
96	ns	ns	ns	ns	ns	ns
108	ns	ns	ns	ns	ns	ns

B — CD4⁺ T cell subsets - LNP transfection

	$T_{naive\ like}$			T_{CM}		T_{EM}
Time (h)	T_{CM}	T_{EM}	T_{EMRA}	T_{EM}	T_{EMRA}	T_{EMRA}
4	ns	ns	ns	ns	ns	ns
8	*	*	*	*	ns	*
24	*	*	**	*	ns	ns
36	*	*	*	ns	ns	ns
48	ns	*	ns	ns	ns	ns
60	ns	ns	ns	ns	ns	****
72	ns	**	ns	ns	ns	ns
84	*	ns	ns	ns	*	**
96	ns	ns	ns	ns	ns	ns
108	ns	ns	ns	ns	ns	ns

D

mock	EP vs. LNP Immunocult	EP vs. LNP CTL	LNP Immunocult vs. LNP CTL
**CD8+**			
$T_{naive\ like}$	ns	ns	ns
T_{CM}	ns	ns	ns
T_{EM}	ns	ns	ns
T_{EMRA}	**	ns	ns
**CD4+**			
$T_{naive\ like}$	ns	ns	ns
T_{CM}	ns	ns	ns
T_{EM}	ns	ns	ns
T_{EMRA}	ns	ns	ns

CD19-CAR+	EP vs. LNP Immunocult	EP vs. LNP CTL	LNP Immunocult vs. LNP CTL
**CD8+**			
$T_{naive\ like}$	ns	ns	ns
T_{CM}	ns	ns	ns
T_{EM}	ns	ns	ns
T_{EMRA}	ns	ns	ns
**CD4+**			
$T_{naive\ like}$	ns	ns	ns
T_{CM}	ns	ns	ns
T_{EM}	ns	ns	ns
T_{EMRA}	ns	ns	ns

CD19-CAR-	EP vs. LNP Immunocult	EP vs. LNP CTL	LNP Immunocult vs. LNP CTL
**CD8+**			
$T_{naive\ like}$	ns	ns	ns
T_{CM}	ns	ns	ns
T_{EM}	ns	ns	ns
T_{EMRA}	ns	ns	ns
**CD4+**			
$T_{naive\ like}$	ns	ns	ns
T_{CM}	ns	ns	ns
T_{EM}	ns	ns	ns
T_{EMRA}	ns	ns	ns

Figure S4: CD19-CAR transfection efficacy in T cell subsets. (**A, B**) CD19-CAR expression levels across memory T-cell subsets within CD4⁺ population following electroporation (**A**) and LNP-mediated mRNA delivery (**B**) over 108 hours post-transfection. (**C**) Frequency of CD19-CAR⁺ T cells 24 hours after mRNA delivery comparing electroporated T cells with LNP-transfected T cells cultured in either CTL medium or ImmunoCult medium. Data represent mean ± SD from n=2 independent donors. (**D**) Memory phenotype characteristics of mock-transfected T cells, CD19-CAR⁺ T cells and CD19-CAR⁻ T cells within CD8⁺ (upper panel) and CD4⁺ (lower panel) populations following electroporation or LNP-mediated mRNA delivery with cultivation in ImmunoCult or CTL medium. Statistical analysis for differences between subpopulations was performed by two-way repeated measures ANOVA with Šidák's multiple comparison test. *ns: not significant, *p<0.05, **p<0.01, ***p<0.001. Data represent mean ± SD from n=4 independent donors, unless stated otherwise.

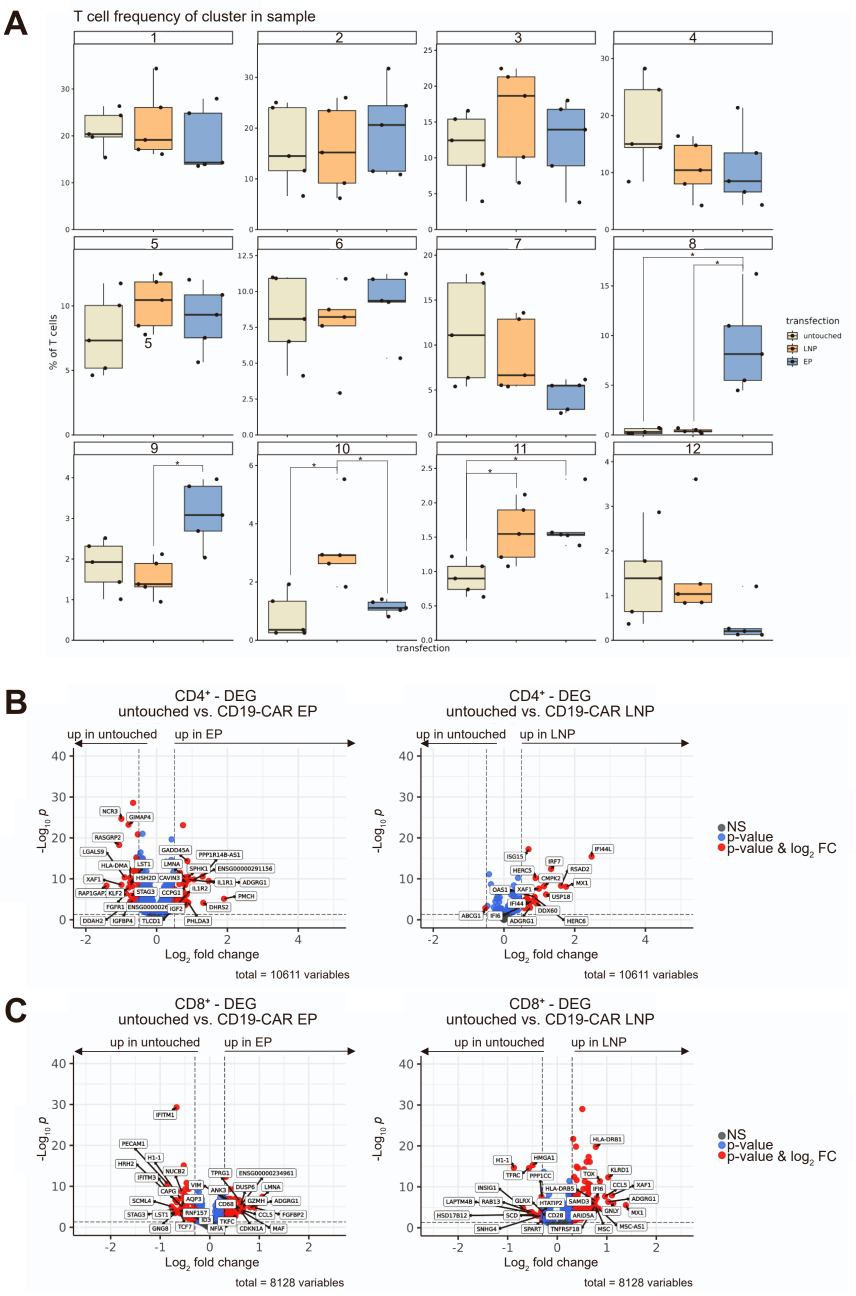

Figure S5: Single-cell transcriptomic changes induced by IVT mRNA delivery methods. (**A**) Abundance of T cell frequency within the different cluster in CD4⁺ and CD8⁺ T cells comparing untouched, LNP-transfected and electroporated CD19-CAR T cells. Data represents n=5 independent donors. Median frequency per donor are plotted. (**B-C**) Volcano plot of differentially expressed genes (DEGs) between LNP and EP transfected CD4⁺ (**B**) and CD8⁺ (**C**) T cells. DE testing was performed using a paired pseudobulk approach using DESeq2. Datapoints for genes with baseMean > 50 are plotted, log2FoldChange threshold set to 0.5 (CD4⁺) and 0.3 (CD8⁺), adjusted p value threshold set to 0.05. Top 15 DEG according to Log2FoldChange above both thresholds are labelled. (**B-C**) box plots showing median and lower and upper hinges correspond to the first and third quartiles. Upper and lower whisker extends from the hinge to the largest/smallest value no further than 1.5 * IQR from the hinge. Data beyond the end of the whiskers are plotted individually. Statistical comparisons of median module scores across groups were performed using the Kruskal–Wallis test, followed by Dunn's post hoc test with Holm adjustment for multiple testing. ns: not significant, *p < 0.05, **p < 0.01, ***p < 0.001, ****p < 0.0001. Data represents data from n=5 independent donors.

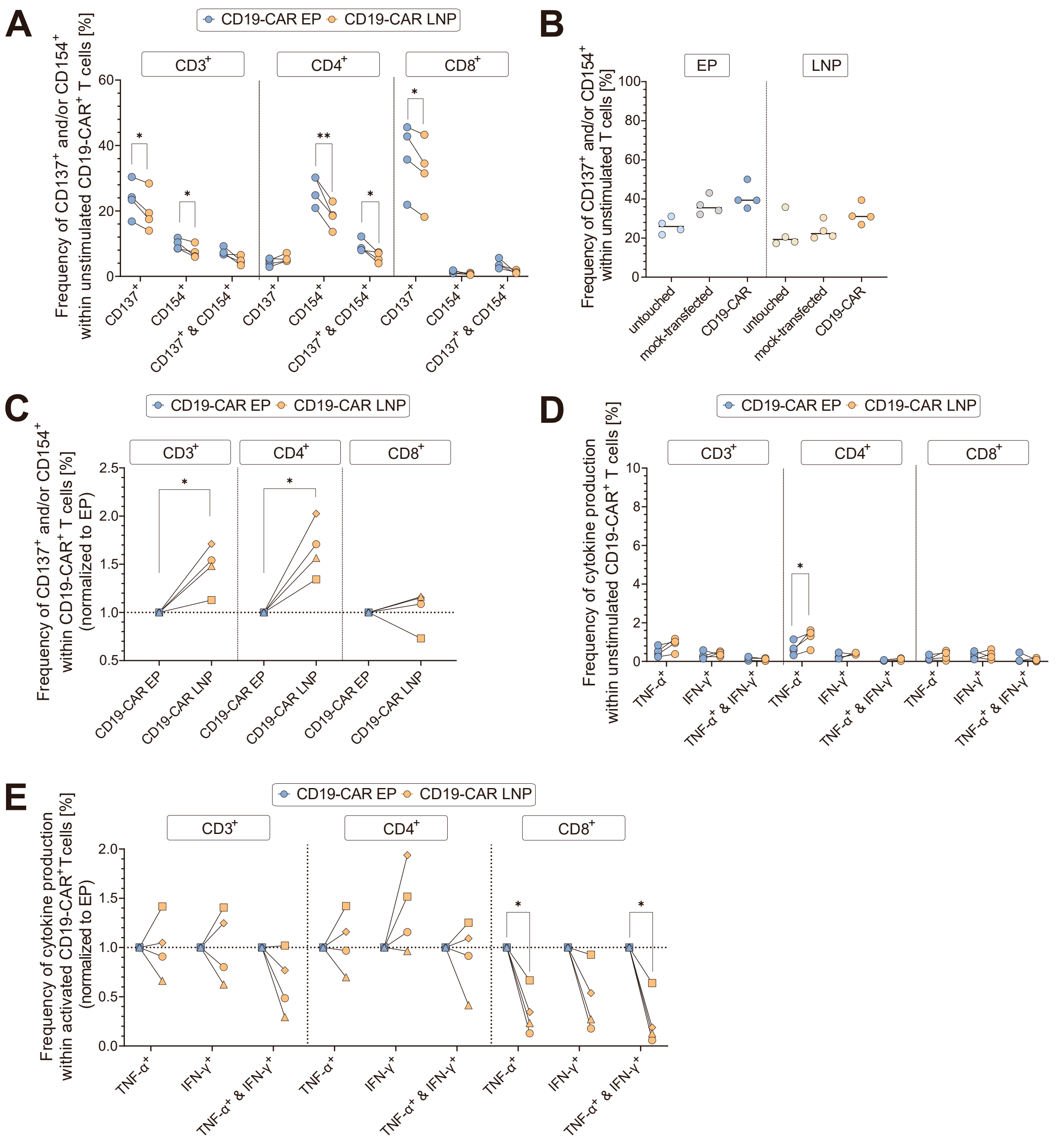

Figure S6: Cytokine production and activation marker expression of CD19-CAR^{+} T cells with and without antigen encounter using CD19^{+} NALM6 target cells. (**A, B**) Flow cytometric analysis of activation marker (CD137^{+} and/or CD154^{+}) within CD3^{+}, CD4^{+} and CD8^{+} T-cell population comparing electroporation and LNP delivery methods in (**A**) unstimulated T cells, (**B**) NALM6-stimulated T cells with background activation subtracted and (**C**) NALM6-stimulated T cells showing the relative increase in frequency of activated T cells for LNP transfection normalized to electroporation. (**D, E**) Flow cytometric analysis of intracellular effector cytokines (IFN-γ^{+} and TNF-α^{+}) within CD3^{+}, CD4^{+} and CD8^{+} T-cell populations comparing electroporation and LNP delivery methods in (**D**) unstimulated T cells and (**E**) NALM6-stimulated T cells showing the relative increase in frequency of activated T cells for LNP transfection normalized to electroporation. Statistical analysis for differences between subpopulations was performed by paired t-test. *ns: not significant, *p<0.05, **p<0.01, ***p<0.001. Data represent n=4 independent donors.

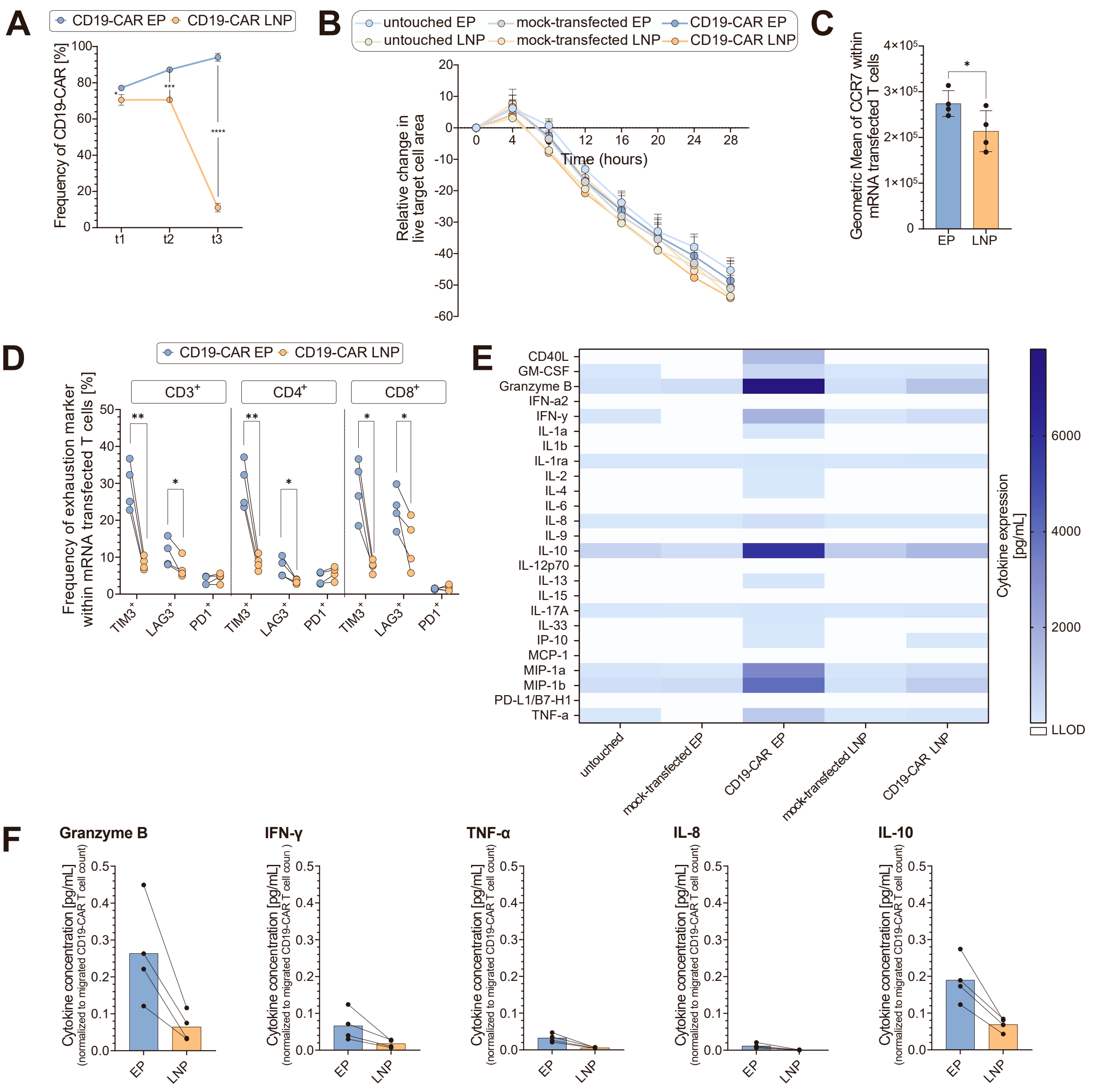

Figure S7: Functional characterization of CD19-CAR$^+$ T cells comparing electroporation and LNP-mediated mRNA delivery methods. (**A**) Flow cytometric analysis over time of CD19-CAR$^+$ frequency in LNP-transfected versus electroporated T-cells at migration assay seeding (t1), killing assay seeding (t2), and post-killing timepoints (t3). (**B**) Cytotoxic efficacy against NALM6 (CD19$^+$ GFP$^+$) target cells in the transwell assay in the absence of CCL21. Killing was assessed over 28 hours using live-cell imaging every 4 hours following spontaneous T cell migration toward medium without CCL21. (**C**) CCR7 expression levels in CD19-CAR T cells at the time of seeding for the killing assay (t2) comparing electroporation versus LNP-generated cells. (**D**) Flow cytometric analysis of exhaustion marker (TIM-3, LAG-3, PD-1) in CD3$^+$, CD4$^+$ and CD8$^+$ population following cytotoxic activity by LNP-transfected and electroporated T cells. (**E**) Cytokine analysis of supernatants collected from the T cell killing assay, measuring 25 cytokines. Data are shown in pg/mL and normalized to CD19-CAR$^+$ frequency of each sample. (**F**) Supernatants from migrated T cell killing assays were collected and analyzed for pro-inflammatory cytokines. Mean levels of Granzyme B, IFN-γ, TNF-α, IL-8 and IL-10 are shown. Normalized to migrated CD19-CAR T cell count for each sample. Statistical analysis for differences between subpopulations was performed using a paired t-test. *ns: not significant, *p<0.05, **p<0.01, ***p<0.001. Data represent mean ± SEM from n=4 independent donors, unless stated otherwise.

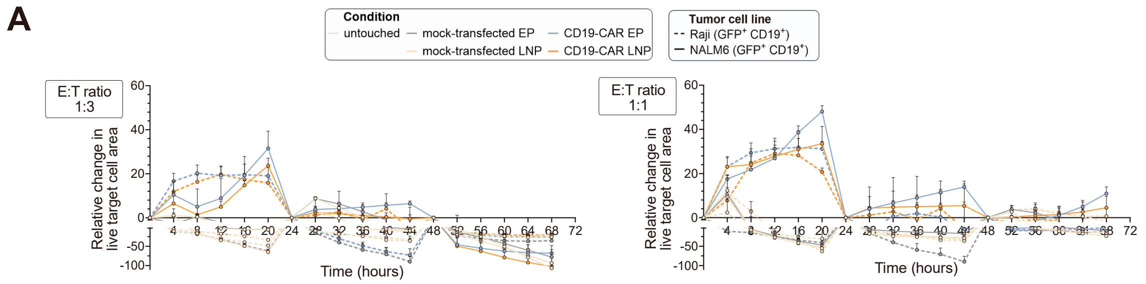

	untouched				mock-transfected EP			CD19-CAR EP		mock-transfected LNP
hours	mock-transfected EP	CD19-CAR EP	mock-transfected LNP	CD19-CAR LNP	CD19-CAR EP	mock-transfected LNP	CD19-CAR LNP	mock-transfected LNP	CD19-CAR LNP	CD19-CAR LNP
4	ns	*	ns	ns	**	ns	*	*	ns	ns
8	ns	ns	ns	ns	ns	ns	ns	*	ns	**
12	ns	*	ns	*	*	ns	*	**	ns	***
16	ns	**	ns	*	*	ns	*	***	ns	**
20	ns	**	ns	*	*	ns	*	***	ns	***
24	ns	ns	ns	ns	ns	ns	ns	ns	ns	ns
28	ns	ns	ns	ns	ns	ns	ns	ns	ns	ns
32	ns	ns	ns	ns	ns	ns	ns	ns	ns	ns
36	ns	ns	ns	ns	ns	ns	ns	ns	ns	ns
40	ns	ns	ns	ns	ns	ns	ns	ns	ns	ns
44	ns	ns	ns	ns	ns	ns	ns	ns	ns	ns
48	ns	ns	ns	ns	ns	ns	ns	ns	ns	ns
52	ns	ns	ns	ns	ns	ns	ns	ns	ns	ns
56	ns	ns	ns	ns	ns	ns	ns	ns	ns	ns
60	ns	ns	ns	ns	ns	ns	ns	ns	ns	ns
64	ns	ns	ns	ns	ns	ns	ns	ns	ns	ns
68	ns	ns	ns	ns	ns	ns	ns	ns	ns	ns
72	ns	ns	ns	ns	ns	ns	ns	ns	ns	ns

	untouched				mock-transfected EP			CD19-CAR EP		mock-transfected LNP
hours	mock-transfected EP	CD19-CAR EP	mock-transfected LNP	CD19-CAR LNP	CD19-CAR EP	mock-transfected LNP	CD19-CAR LNP	mock-transfected LNP	CD19-CAR LNP	CD19-CAR LNP
4	ns	ns	ns	ns	ns	ns	ns	*	ns	ns
8	ns	***	ns	**	**	ns	**	***	ns	**
12	ns	**	ns	**	***	ns	***	****	ns	****
16	ns	*	ns	*	***	ns	****	****	ns	****
20	ns	*	ns	*	****	ns	****	****	ns	****
24	ns	ns	ns	ns	ns	ns	ns	ns	ns	ns
28	ns	ns	ns	ns	ns	ns	ns	ns	ns	*
32	ns	ns	ns	**	ns	ns	ns	ns	ns	*
36	ns	ns	ns	**	ns	ns	ns	ns	ns	**
40	ns	*	ns	***	ns	ns	ns	*	ns	**
44	ns	ns	ns	**	ns	ns	ns	ns	ns	*
48	ns	ns	ns	ns	ns	ns	ns	ns	ns	ns
52	ns	**	ns	ns	ns	ns	ns	*	ns	ns
56	ns	ns	ns	ns	ns	ns	ns	ns	ns	ns
60	ns	ns	ns	ns	ns	ns	ns	ns	ns	ns
64	ns	ns	ns	ns	ns	ns	ns	ns	ns	ns
68	ns	ns	ns	ns	ns	ns	ns	ns	ns	ns
72	ns	ns	ns	ns	*	ns	ns	ns	ns	ns

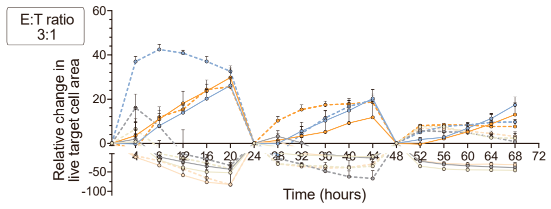

	untouched				mock-transfected EP			CD19-CAR EP		mock-transfected LNP
hours	mock-transfected EP	CD19-CAR EP	mock-transfected LNP	CD19-CAR LNP	CD19-CAR EP	mock-transfected LNP	CD19-CAR LNP	mock-transfected LNP	CD19-CAR LNP	CD19-CAR LNP
4	ns	ns	ns	ns	ns	ns	ns	ns	ns	ns
8	ns	ns	ns	ns	ns	ns	ns	ns	ns	ns
12	ns	ns	ns	ns	ns	ns	ns	*	ns	ns
16	ns	*	ns	ns	*	ns	ns	*	ns	ns
20	ns	**	ns	*	**	ns	*	*	ns	*
24	ns	**	ns	**	**	ns	**	*	ns	ns
28	ns	ns	ns	ns	ns	ns	ns	ns	ns	ns
32	ns	ns	ns	ns	ns	ns	ns	ns	ns	ns
36	ns	ns	ns	*	ns	ns	ns	ns	ns	ns
40	ns	*	ns	*	ns	ns	ns	*	ns	*
44	ns	ns	ns	ns	ns	ns	ns	*	ns	*
48	ns	ns	ns	ns	*	ns	ns	**	ns	**
52	ns	ns	ns	ns	ns	ns	ns	ns	ns	ns
56	ns	ns	ns	ns	ns	ns	ns	ns	ns	ns
60	ns	ns	ns	ns	ns	ns	ns	ns	ns	ns
64	ns	ns	ns	ns	ns	ns	ns	ns	ns	ns
68	ns	ns	ns	ns	ns	ns	ns	ns	ns	ns
72	ns	ns	ns	ns	ns	ns	ns	ns	ns	ns

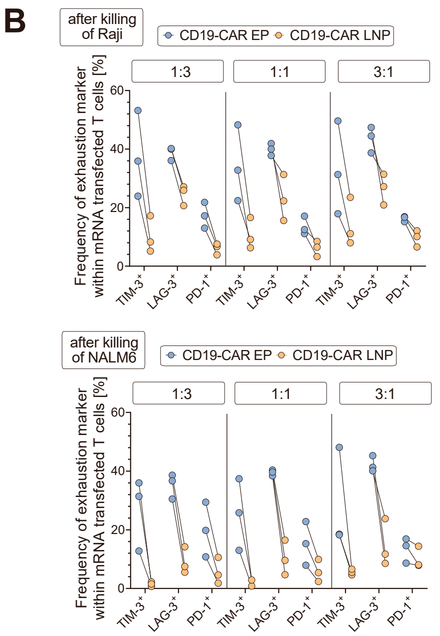

Figure S8: Tumor rechallenge assay comparing cytotoxic capacity of CD19-CAR⁺ T cells generated by electroporation versus LNP-mediated mRNA delivery. (**A**) Cytotoxic efficacy against NALM6 and Raji tumor cell rechallenge (both CD19⁺ GFP⁺) by untouched, mock-transfected and CD19-CAR⁺ T cells generated via electroporation or LNP transfection. Killing was observed over 68 hours using live-cell imaging every 4 hours. Fresh target cells were added every 24 hours at effector-to-target (E:T) ratio of 1:3, 1:1 and 3:1 based on the initial CAR T cell number seeded. (**B**) Flow cytometric analysis of exhaustion marker expression (TIM-3, LAG-3, PD-1) in CD3⁺, CD4⁺ and CD8⁺ T-cell populations following rechallenged cytotoxic activity of LNP-transfected T-cells normalized to electroporated T-cells over 68 hours. Statistical analysis was performed on pooled data from both B-cell lines (NALM6 and Raji) using two-way repeated measures ANOVA with Šidák's multiple comparison test. Data represent mean ± SEM from n=3 independent donors. *ns: not significant, *p<0.05, **p<0.01, ***p<0.001. Data represent mean ± SEM from n=3 independent donors.

Table S1: Coding sequences used for integration into the plasmid vector template pRNA2-(A)128. Coding sequences for CD19-CAR, CCR7, CXCR3, and EGFP are shown 5' to 3'. Sequences were designed in silico and synthesized by Integrated DNA Technologies for InFusion or conventional cloning.

**Construct**	**Sequence**
**CD19-CAR**	5'-<br>ATGGCTCTTCCTGTGACTGCCCTTCTGCTGCCCCTGGCTCTCCTGCTCCATGCTGCCCGGCCAga<br>gcaaaaacttatctctgaagaggacctcGACATCCAGATGACACAGACAACCAGCAGCCTCAGCGCCA<br>GCCTGGGGGACAGAGTGACCATTAGCTGCCGGGCCTCTCAGGACATCAGCAAATACCTGAAC<br>TGGTACCAGCAGAAACCAGATGGCACTGTCAAGCTGCTGATTTACCACACCTCCAGGCTCCAC<br>AGCGGCGTGCCCAGTCGCTTCAGCGGCAGTGGGAGCGGGACAGATTATTCCCTCACAATCTC<br>CAACCTGGAGCAGGAAGATATTGCCACATACTTCTGCCAGCAAGGCAACACCCTGCCATACAC<br>ATTTGGAGGCGGCACCAAATTGGAGATCACCGGCGGTGGTGGATCTGGAGGAGGGGGCAGC<br>GGAGGTGGCGGCTCTGAGGTGAAACTGCAGGAGAGTGGCCCTGGCCTGGTGGCTCCCAGCC<br>AGAGCCTTTCTGTCACCTGCACCGTGTCTGGGGTGTCCCTGCCTGACTATGGAGTCTCCTGGA<br>TCCGGCAGCCTCCAAGAAAAGGACTGGAATGGCTGGGCGTCATCTGGGGAAGTGAGACCAC<br>CTACTATAATTCAGCCCTCAAGTCCCGGCTCACCATCATTAAGGACAACTCCAAATCCCAGGTG<br>TTCCTGAAGATGAATTCTCTCCAGACTGATGACACAGCCATCTACTACTGTGCCAAGCATTATT<br>ATTACGGCGGGTCCTATGCCATGGACTACTGGGGCCAGGGGACCAGTGTCACTGTTTCTTCTA<br>TAGAAGTAATGTATCCCCCTCCCTACTTGGACAACGAGAAATCTAACGGCACAATCATACACG<br>TTAAGGGCAAGCATCTGTGTCCCTCCCCTCTTTTCCCCGGACCGTCTAAGCCATTTTGGGTCCT<br>CGTGGTGGTCGGGGGTGTGCTGGCCTGCTACAGCTTGCTGGTCACAGTGGCCTTCATCATCTT<br>CTGGGTGCGCTCCAAGAGGAGCCGGCTGCTTCACAGTGATTACATGAACATGACCCCCAGGA<br>GGCCAGGACCCACCAGGAAGCACTACCAGCCCTACGCTCCCCCGCGGGACTTTGCTGCTTACC<br>GCAGCAGGGTCAAATTTTCTAGATCTGCAGATGCGCCGGCCTATCAAcaaggccagaaccagctcT<br>ATAACGAGCTCAATCTAGGACGAAGAGAGGAGTACGATGTTTTGGACAAGAGACGTGGCCG<br>GGACCCTGAGATGGGGGGAAAGCCGAGAAGGAAGAACCCTCAGGAAGGCCTGTACAATGA<br>ACTGCAGAAAGATAAGATGGCGGAGGCCTACAGTGAGATTGGGATGAAAGGCGAGCGCCG<br>GAGGGGCAAGGGGCACGATGGCCTTTACCAGGGTCTCAGTACAGCCACCAAGGACACCTAC<br>GACGCCCTTCACATGCAGGCCCTGCCCCCTCGCTAA -3'
**EGFP**	5'-<br>ATGGTGAGCAAGGGCGAGGAGCTGTTCACCGGGGTGGTGCCCATCCTGGTCGAGCTGGACG<br>GCGACGTAAACGGCCACAAGTTCAGCGTGTCCGGCGAGGGCGAGGGCGATGCCACCTACGG<br>CAAGCTGACCCTGAAGTTCATCTGCACCACCGGCAAGCTGCCCGTGCCCTGGCCCACCCTCGT<br>GACCACCCTGACCTACGGCGTGCAGTGCTTCAGCCGCTACCCCGACCACATGAAGCAGCACG<br>ACTTCTTCAAGTCCGCCATGCCCGAAGGCTACGTCCAGGAGCGCACCATCTTCTTCAAGGACG<br>ACGGCAACTACAAGACCCGCGCCGAGGTGAAGTTCGAGGGCGACACCCTGGTGAACCGCAT<br>CGAGCTGAAGGGCATCGACTTCAAGGAGGACGGCAACATCCTGGGGCACAAGCTGGAGTAC<br>AACTACAACAGCCACAACGTCTATATCATGGCCGACAAGCAGAAGAACGGCATCAAGGTGAA<br>CTTCAAGATCCGCCACAACATCGAGGACGGCAGCGTGCAGCTCGCCGACCACTACCAGCAGA<br>ACACCCCCATCGGCGACGGCCCCGTGCTGCTGCCCGACAACCACTACCTGAGCACCCAGTCCG<br>CCCTGAGCAAAGACCCCAACGAGAAGCGCGATCACATGGTCCTGCTGGAGTTCGTGACCGCC<br>GCCGGGATCACTCTCGGCATGGACGAGCTGTACAAGTAA -3'
**CCR7**	5'-<br>ATGGACCTGGGGAAACCAATGAAAAGCGTGCTGGTGGTGGCTCTCCTTGTCATTTTCCAGGTA<br>TGCCTGTGTCAAGATGAGGTCACGGACGATTACATCGGAGACAACACCACAGTGGACTACAC<br>TTTGTTCGAGTCTTTGTGCTCCAAGAAGGACGTGCGGAACTTTAAAGCCTGGTTCCTCCCTATC<br>ATGTACTCCATCATTTGTTTCGTGGGCCTACTGGGCAATGGGCTGGTCGTGTTGACCTATATCT<br>ATTTCAAGAGGCTCAAGACCATGACCGATACCTACCTGCTCAACCTGGCGGTGGCAGACATCC

	TCTTCCTCCTGACCCTTCCCTTCTGGGCCTACAGCGCGGCCAAGTCCTGGGTCTTCGGTGTCCA<br>CTTTTGCAAGCTCATCTTTGCCATCTACAAGATGAGCTTCTTCAGTGGCATGCTCCTACTTCTTT<br>GCATCAGCATTGACCGCTACGTGGCCATCGTCCAGGCTGTCTCAGCTCACCGCCACCGTGCCC<br>GCGTCCTTCTCATCAGCAAGCTGTCCTGTGTGGGCATCTGGATACTAGCCACAGTGCTCTCCAT<br>CCCAGAGCTCCTGTACAGTGACCTCCAGAGGAGCAGCAGTGAGCAAGCGATGCGATGCTCTC<br>TCATCACAGAGCATGTGGAGGCCTTTATCACCATCCAGGTGGCCCAGATGGTGATCGGCTTTC<br>TGGTCCCCCTGCTGGCCATGAGCTTCTGTTACCTTGTCATCATCCGCACCCTGCTCCAGGCACG<br>CAACTTTGAGCGCAACAAGGCCATCAAGGTGATCATCGCTGTGGTCGTGGTCTTCATAGTCTT<br>CCAGCTGCCCTACAATGGGGTGGTCCTGGCCCAGACGGTGGCCAACTTCAACATCACCAGTA<br>GCACCTGTGAGCTCAGTAAGCAACTCAACATCGCCTACGACGTCACCTACAGCCTGGCCTGCG<br>TCCGCTGCTGCGTCAACCCTTTCTTGTACGCCTTCATCGGCGTCAAGTTCCGCAACGATCTCTT<br>CAAGCTCTTCAAGGACCTGGGCTGCCTCAGCCAGGAGCAGCTCCGGCAGTGGTCTTCCTGTC<br>GGCACATCCGGCGCTCCTCCATGAGTGTGGAGGCCGAGACCACCACCACCTTCTCCCCATAG-<br>3'
**CXCR3**	5'-<br>ATGGTGTTGGAAGTCAGCGACCACCAAGTTCTTAATGATGCTGAGGTCGCCGCACTTCTCGAA<br>AACTTCTCTAGCTCCTATGACTACGGAGAAAACGAAAGTGATTCTTGCTGCACGAGTCCACCG<br>TGTCCCCAAGATTTCAGCCTCAACTTCGACCGAGCCTTCTTGCCCGCGCTCTACTCCTTGCTGTT<br>CTTGCTCGGTCTTCTCGGGAACGGCGCAGTGGCGGCGGTTCTTCTTTCCAGACGAACCGCCCT<br>GTCATCTACGGACACATTCCTGTTGCATCTCGCGGTTGCTGACACACTTCTGGTTTTGACCCTG<br>CCTCTTTGGGCAGTAGACGCCGCCGTACAATGGGTGTTCGGCAGTGGCTTGTGCAAGGTAGC<br>AGGTGCACTGTTCAACATTAACTTCTATGCCGGAGCCCTTCTCTTGGCGTGTATTAGCTTCGAC<br>AGATATCTCAATATTGTCCACGCGACTCAACTCTACCGCCGGGGACCCCCAGCACGCGTTACG<br>CTTACATGCCTTGCCGTATGGGGCCTTTGTCTCCTTTTCGCGTTGCCAGATTTTATCTTCCTCTC<br>CGCACACCACGACGAAAGGTTGAACGCAACACACTGTCAGTATAACTTTCCCCAAGTGGGGC<br>GCACGGCCCTGCGGGTTTTGCAGCTTGTAGCAGGGTTCTTGTTGCCCCTTCTTGTGATGGCAT<br>ATTGCTATGCTCATATACTGGCTGTACTTTTGGTAAGTCGCGGTCAGCGACGCCTTCGCGCCAT<br>GCGACTTGTAGTGGTTGTAGTGGTTGCCTTTGCCCTGTGCTGGACCCCGTATCACCTCGTGGT<br>GCTTGTAGATATTCTTATGGACCTGGGCGCCCTCGCTCGGAACTGCGGGCGGGAGTCCAGGG<br>TGGATGTGGCGAAATCTGTGACGAGTGGCCTTGGCTACATGCATTGTTGCCTCAATCCCCTGT<br>TGTATGCTTTTGTTGGGGTTAAGTTCAGGGAAAGAATGTGGATGCTTCTTCTTAGGTTGGGCT<br>GCCCCAACCAGAGAGGACTGCAGCGGCAGCCTTCCAGCTCTCGCCGCGACAGCTCATGGTCA<br>GAAACGAGCGAGGCTAGCTATAGTGGCTTGTAG-3'

Table S2: Primer sequences used for integration of coding sequences into plasmid vector template pRNA2-(A)128 and for IVT amplification. Primer sequences binding to CD19-CAR, CXCR3, and IVT construct are shown 5' to 3'. Sequences were designed in silico and synthesized by Integrated DNA Technologies for InFusion or conventional cloning.

Target	Forward/Reverse	Primer Sequence
pRNA2	forward	5'-AGCGGCCGCAGCT-3'
pRNA2	reverse	5'-GGTGGCGACCGGTGG-3'
CD19-CAR	forward	5'-CCACCGGTCGCCACCATGGCTCTTCCTGTGACTGC-3'
CD19-CAR	reverse	5'-AGCTGCGGCCGCTGGCAACTAGAAGGCACAG-3'
CXCR3	forward	5'-CCACCGGTCGCCACCATGGTGTTGGAAGTCAGCGAC-3'
CXCR3	reverse	5'-CGAGCTGCGGCCGCTCTACAAGCCACTATAGCTAGCCTC-3'
T7 promotor	forward	5'-GCTCTTAAGGCTAGAGTACTTAATACGACTCACTATAGGGAG-3'
poly(A) tail	reverse	5'-TTTTTTTTTTTTTTTTTTTTTTTTTTTTTTTTTTTTTTTTTTTTTTTTTTTTTT<br>TTTTTTTTTTTTTTTTTTTTTTTTTTTTTTTTTTTTTTTTTTTTTTTTTTTTTT<br>TTTTTTTTTTTTTTTTTTTTTTGTTTAAACATTTAAATGCAAT-3'
